# Isolation and Molecular Analysis of a Novel *Neorickettsia* Species That Causes Potomac Horse Fever

**DOI:** 10.1128/mBio.03429-19

**Published:** 2020-02-25

**Authors:** Omid Teymournejad, Mingqun Lin, Hannah Bekebrede, Ahmed Kamr, Ramiro E. Toribio, Luis G. Arroyo, John D. Baird, Yasuko Rikihisa

**Affiliations:** a Laboratory of Molecular, Cellular, and Environmental Rickettsiology, Department of Veterinary Biosciences, College of Veterinary Medicine, The Ohio State University, Columbus, Ohio, USA; b Department of Veterinary Clinical Sciences, College of Veterinary Medicine, The Ohio State University, Columbus, Ohio, USA; c Department of Clinical Studies, Ontario Veterinary College, University of Guelph, Guelph, Ontario, Canada; Brigham and Women’s Hospital

**Keywords:** Koch’s postulates, *Neorickettsia* species, Potomac horse fever, major antigen, obligatory intracellular, phylogenetic analysis, whole-genome sequence

## Abstract

Potomac horse fever (PHF), a severe and frequently fatal febrile diarrheal disease, has been known to be caused only by Neorickettsia risticii, an endosymbiont of digenean trematodes. Here, we report the cell culture isolation of a new *Neorickettsia* species found in two locations in eastern Ontario, Canada, in 2016 and 2017 (in addition to 10 variable strains of N. risticii) from *N. risticii* PCR-negative horses with clinical signs of PHF. Gene sequences of 16S rRNA and the major surface antigen P51 of this new *Neorickettsia* species were distinct from those of all previously characterized *N. risticii* strains and *Neorickettsia* species, except for those from an uncharacterized *Neorickettsia* species culture isolate from a horse with PHF in northern Ohio in 1991. The new *Neorickettsia* species nonetheless had the characteristic intramolecular repeats within strain-specific antigen 3 (Ssa3), which were found in all sequenced Ssa3s of *N. risticii* strains. Experimental inoculation of two naive ponies with the new *Neorickettsia* species produced severe and subclinical PHF, respectively, and the bacteria were reisolated from both of them, fulfilling Koch’s postulates. Serological assay titers against the new *Neorickettsia* species were higher than those against *N. risticii*. Whole-genome sequence analysis of the new *Neorickettsia* species revealed unique features of this bacterium compared with *N. risticii*. We propose to classify this new bacterium as Neorickettsia finleia sp. nov. This finding will improve the laboratory diagnosis of and vaccine for PHF, environmental risk assessment of PHF, and understanding of PHF pathogenesis and *Neorickettsia* biology in general.

## Abstract (precis)

Despite the detection of *Neorickettsia* species DNA sequences in various trematode species and their hosts, only three *Neorickettsia* species have been cell culture isolated and whole-genome sequenced and are known to infect mammals and/or cause disease. The molecular mechanisms that enable the obligatory intracellular bacterium *Neorickettsia* to colonize trematodes and to horizontally transmit from trematodes to mammals, as well as the virulence factors associated with specific mammalian hosts, are unknown. Potomac horse fever (PHF) is a severe and acute systemic infectious disease of horses, with clinical signs that include diarrhea. Neorickettsia risticii is the only known bacterial species that causes PHF. Ingestion of insects harboring *N. risticii*-infected trematodes by horses leads to PHF. Our discovery of a new *Neorickettsia* species that causes PHF and whole-genome sequence analysis of this bacterium will improve laboratory diagnosis and vaccine development for PHF and will contribute to our understanding of *Neorickettsia* ecology, pathogenesis, and biology.

## INTRODUCTION

*Neorickettsia* spp. are obligatory intracellular bacteria of digenean trematodes, a kind of fluke, which are transmitted through all developmental stages of the trematodes and vertically through generations of trematodes (1–4). *Neorickettsia* is present in various trematode species, including Fasciola hepatica, the liver fluke of humans, cattle, and sheep, and hosts of unknown trematodes worldwide (4–11). Considering the broad distribution of *Neorickettsia* spp. in nature, it is important to understand how these pathogenic species can jump from trematodes into vertebrates and cause illnesses, as such an understanding will improve laboratory diagnosis, treatment, and prevention. Currently, PCR, serology, and cell culture isolation are used to detect and characterize these bacteria. Among these techniques, only cell culture isolation provides definitive proof for the presence of live *Neorickettsia* bacteria in an isolate, and isolation of *Neorickettsia* from the environment is needed if we are to increase our understanding of its life cycle for use in basic and translational research (12). Because difficult and cumbersome techniques and specific equipment are required to culture and isolate these bacteria, only three well-defined *Neorickettsia* species, N. risticii (formerly Ehrlichia risticii), N. sennetsu (formerly Ehrlichia sennetsu and Rickettsia sennetsu), and N. helminthoeca (type species), and one additional *Neorickettsia* species denoted as the fluke Stellantchasmus falcatus agent (SF agent) have been stably culture isolated (13–21). *N. risticii*, *N. sennetsu*, and *N. helminthoeca* are horizontally transmitted to definitive or accidental mammalian hosts of trematodes and subsequently cause severe acute or chronic disease (1, 12). *N. risticii* causes Potomac horse fever (PHF) in North and South America, *N. helminthoeca* causes salmon-poisoning disease in North and South American canids, and *N. sennetsu* causes human Sennetsu neorickettsiosis in Japan and Southeast Asia (12, 22, 23). Hirose, the SF agent strain, was originally isolated in Japan in 1962 from encysted S. falcatus metacercariae in gray mullet fish (19). The strain Hirose was stably cultured and molecularly characterized as a *Neorickettsia* sp. closely related to *N. risticii* but distinct from *N. helminthoeca*, which infects Nanophyetus salmincola trematodes that also encyst in fish (20, 21). Experimental inoculation of the strain Hirose causes mild clinical signs in dogs but severe splenomegaly and lymphadenopathy in mice (19). A second strain of the SF agent was molecularly characterized following culture isolation from a dog that was experimentally fed trout caught in Oregon (21). The dog showed severe clinical signs resembling salmon-poisoning disease.

PHF is an acute, severe, or fatal systemic disease of horses characterized by fever, depression, anorexia, dehydration, watery diarrhea, laminitis, and/or abortion, which can inflict great economical and emotional losses (14, 24, 25). PHF typically occurs in the warm-weather months of early to late summer (14, 25). PHF is frequently diagnosed in the United States and is identified only occasionally in Brazil, Uruguay, and Europe (24). In Canada, there has been an increase in the confirmation of the diagnosis of PHF by equine veterinarians (26–28). Culture isolation of *N. risticii* has been reported only for horses suffering from PHF in the United States and Canada (14, 15, 29–31). The only effective treatment for PHF is the administration of oxytetracycline during the early stages of the disease (24). Although a vaccine against PHF has been developed, *N. risticii* continues to cause disease despite horses being vaccinated. The lack of protection is likely due to insufficient immunological responses and antigenic variations (29, 32–34).

The trematode host of *N. risticii* in the eastern United States was identified morphologically and molecularly defined as the digenetic trematode Acanthatrium oregonense (1, 35). A. oregonense has a complex life cycle involving miracidia and sporocysts in its snail host (Elimia virginica), free-swimming cercariae, metacercariae in aquatic insects (caddisflies and mayflies), and adults that lay eggs in the intestinal lumen of insectivorous bats (1, 21, 23, 35, 36). Upon ingestion of *N. risticii*-infected metacercarial trematodes within their insect hosts, *N. risticii* is horizontally transmitted from the trematodes to horses, and the bacterium then replicates within inclusion bodies inside monocytes, macrophages, mast cells, and intestinal epithelial cells (24, 25, 31, 37–39).

Although PHF was described as early as 1924 in Ontario, Canada, by Frank Schofield (40), the first culture isolation of *N. risticii* in Canada was in 2015, involving a contemporary *N. risticii* strain that had caused PHF (30). For the study reported here, we isolated *Neorickettsia* from cultures of the blood of 12 horses in Ontario, Canada, that exhibited clinical signs of PHF, molecularly characterized the isolates in terms of currently prevalent strains and species, analyzed the whole genome sequence, and tested a novel isolate in naive ponies by Koch’s postulates.

## RESULTS

### Horses with PHF clinical signs evaluated in the present study.

**FIG 1:**
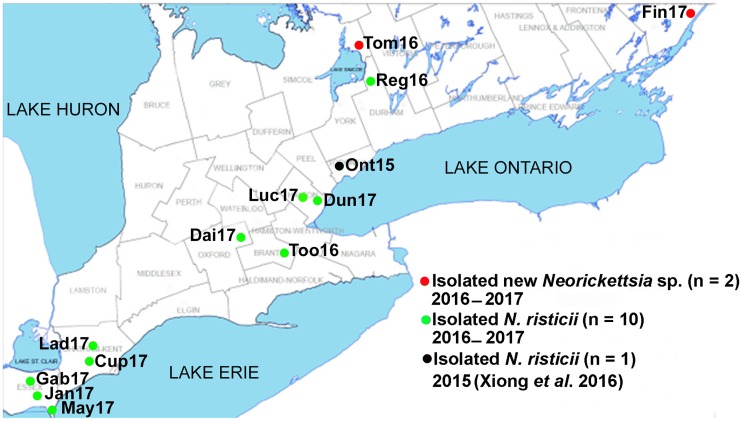
Geographic locations of the horses with PHF, blood cultures of which yielded new *Neorickettsia* sp. or *N. risticii* strains during 2015, 2016, and 2017 for this study. Positive PHF cases identified by the isolation of *N. risticii* strains and new *Neorickettsia* species upon culture are shown. The black dot shows a 2015 culture isolate (30). Strain names are shown next to each dot.

A total of 12 horses were evaluated in the present study. Of those horses, Lad17, Cup17, Gab17, Jan17, and May17 resided near Lake St. Clair and Lake Erie in western Ontario; Dai17, Luc17, Too16, and Dun17 resided near the junction of Lake Ontario and Lake Erie; Reg16 and Tom15 resided near Lake Simcoe; and Fin17 resided near the junction of Lake Ontario and the St. Lawrence River (Fig. 1). The horses showed typical clinical signs of PHF, including fever (5/12), depression (11/12), anorexia (11/12), diarrhea (9/12), abnormal mucous membrane color and increased capillary refill time (10/12), dehydration (10/12), laminitis (2/12), and death/euthanasia (2/12) although not colic (0/12) (Table 1). Two of the horses (Fin17 and Dai17) had been vaccinated with a commercial vaccine (equine Potomavac; Merial Canada, Inc., Baie d’Urfé, QC, Canada) during the spring of 2017 but became ill from PHF the following summer. Real-time PCR tests of 85 bp of the 16S rRNA gene of *N. risticii* (41) were positive for the blood (7/11) and feces (8/12) of the horses, and three horses (Fin17, Tom16, and Luc17) were negative in both blood and feces (Table 1). *Neorickettsia* isolates were obtained from cultures of blood preparations from all 12 horses after 15 to 30 days of culture.

**TABLE 1:** Clinical signs, treatment outcome, and vaccination status of 12 horses from which *Neorickettsia* species were isolated

Horse ID^*a*^	Sex^*b*^	Age	Stabled at night	Sick (days)^*c*^	PHF vaccinated	Depression	Anorexia	Fever	Diarrhea^*d*^	Mucous membranes^*e*^	Laminitis^*f*^	PCR^*h*^		Treatment outcome^*g*^
												Blood	Feces	
Fin17	MC	3	No	1	Yes	Yes	Yes	No	Profuse projectile	Pink	No	−	−	Full recovery
Tom16	MC	8	No	2	No	No	Yes	Yes	Moderate	Dark pink	No	−	−	Full recovery
May17	F	6	No	2	No	Yes	Yes	No	Severe profuse projectile	Purple toxic line	No	−	+	Died day 2
Luc17	F	7	Yes	1	No	Yes	Yes	No	Impaction to profuse	Pink initially	No	−	−	Full recovery
Cup17	F	26	Yes	3	No	Yes	Yes	Yes	No	Toxic line	No	+	+	Full recovery
Lad17	F	5	No	<24 h	No	Yes	No	Yes	Yes	Pink	No	+	+	Full recovery
Dun17	M	4	Yes	7	No	Yes	Yes	No	Profuse projectile	Purple toxic line	Yes	+	+	Euthanized day 8
Jan17	F	12	Yes	2	No	Yes	Yes	Yes	No	Pale pink toxic line	No	NS^*i*^	−	Full recovery
Gab17	MC	9	Yes	1	No	Yes	Yes	Yes	No	Pale pink	No	+	+	Full recovery
Dai17	F	10	Yes, 45 days	2	Yes	Yes	Yes	No	Watery	Dark pink toxic line	No	+	+	Full recovery
Too16	MC	7	Yes	3	No	Yes	Yes	Yes	Mild on admit	Toxic line	Yes	+	+	Full recovery
Reg16	F	22	Yes	3	No	Yes	Yes	No	Yes, watery	Brick red	No	+	+	Full recovery

a Horse as well as *Neorickettsia* strain ID. The last two numbers in each ID indicate the year of *Neorickettsia* species isolation.

b MC, male castrated; F, female; M, male.

c The number of days the horse was observed to be sick by the owner before the attending veterinarian first examined the horse and collected blood samples.

d Nature of diarrhea: mild diarrhea (softer than normal), moderate (“cow pie”), severe (watery, profuse, projectile).

e Color of mucous membranes (buccal and conjunctival). A toxic line is a dark line at the gum line of the incisor teeth, which is interpreted as a sign of toxemia, dehydration, and other abnormal circulatory states. In PHF cases, this is interpreted as a sign of enterotoxaemia and severe dehydration.

f Hoof inflammation/pain.

g All horses were treated with oxytetracycline at 6.6 mg/kg of body weight, intravenously every 24 h for 5 days.

h Performed at OVC-VTH.

i NS, blood sample not submitted.

### P51 sequence analysis.

**FIG 2:**
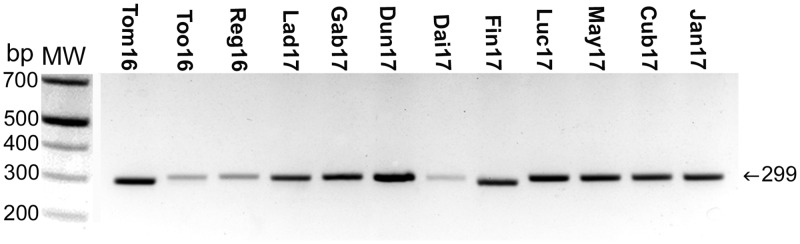
PCR amplification of *Neorickettsia* P51 external loop 2 DNA sequences from the 12 *Neorickettsia* culture isolates. DNA samples from the culture isolates derived from the blood of the infected horses were subjected to PCR using the *Neorickettsia* P51 external loop 2 region and the primers 51K-F7 and 51K-R5. Agarose gel electrophoresis of PCR amplified products was performed. The expected PCR product size of *N. risticii* was 299 bp. Note the smaller products amplified from Tom16 and Fin17 DNAs. MW, standard molecular size markers.

**FIG 3:**
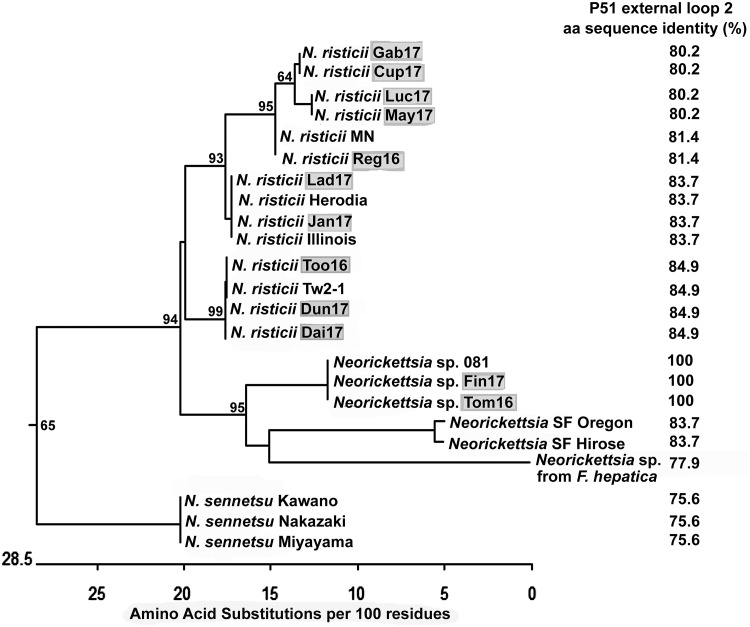
Phylogenetic tree of *Neorickettsia* P51 external loop 2 amino acid sequences encoded in the genomes of the 12 *Neorickettsia* culture isolates described here. Positions of the deduced loop 2 amino acid sequences (86 residues) from P51 of these 12 *Neorickettsia* species and *N. risticii* culture isolates (highlighted in gray) and those of closely related *Neorickettsia* spp. are shown on the phylogenetic tree. Note the clustering of Fin17 and Tom16 with *Neorickettsia* sp. 081 sequences, which are separated from the *N. risticii* clades. Bootstrap values (>50) for 1,000 replicates are shown at each branch point. Percent values to the right of the strain names indicate amino acid identity values obtained in comparison with strain Fin17^T^.

P51 is a 51-kDa *Neorickettsia* outer membrane protein composed of a transmembrane β-barrel domain with 18 transmembrane segments and an extracellular domain containing nine loops (34). The P51 amino acid sequence, in particular, the surface-exposed loop 2, is highly variable among isolated *N. risticii* strains and thus is suitable for initial confirmation and comparison of *Neorickettsia* isolates (21, 34, 42). To characterize the 12 Canadian PHF isolates, we amplified the P51 loop 2 DNA fragment by using PCR with loop 2-specific primers designed for conserved sequences of multiple *Neorickettsia* species (see Table S1 in the supplemental material) (34). All 12 isolates were PCR positive for P51 loop 2 (Fig. 2). Interestingly, two of the PCR products (those of the Tom16 and Fin17 isolates) were similar in size but were slightly smaller than those of the other 10 isolates (Fig. 2). All 12 amplified products were sequenced. The deduced loop 2 amino acid sequences (defined as the 86 residues between positions 126 and 211 of P51 from *N. risticii* Illinois, which had been isolated from a horse in Maryland) for our 12 newly cultured specimens were aligned with those of several *N. risticii* strains from horses that had resided in Minnesota, Kentucky, Pennsylvania, and Maryland. The aligned sequences were used for phylogenetic analysis. The Ohio 081 strain (*Neorickettsia* sp. 081) isolated from a horse with PHF in Finley, OH, in 1991 (29, 43, 44); three *N. sennetsu* strains from Japan, the SF agents from Japan (Hirose) and the United States (Oregon); and the *Neorickettsia* sp. from F. hepatica were also included in the analysis as they are closely related to *N. risticii*. Surprisingly, for Fin17 and Tom16, their P51 loop 2 amino acid sequences were identical to that of *Neorickettsia* sp. 081 but were distinct from those of the *N. risticii* strains (Fig. 3 and Fig. S1A and B). The P51 loop 2 amino acid sequences of Fin17, Tom16, and *Neorickettsia* sp. 081 were clustered next to the Oregon and Hirose SF agents rather than with the *N. risticii* strains (Fig. 3). The P51 loop 2 amino acid sequence of *Neorickettsia* sp. from *F. hepatica* was also clustered next to the SF agents but was only remotely related to the loop 2 amino acid sequences of the SF agents owing to multiple nonsynonymous mutations (Fig. S1A). The remaining 10 P51 loop 2 amino acid sequences from the Canadian isolates clustered with sequences from strains isolated in the eastern and Midwestern United States, as follows. Five of the Canadian strains clustered with the *N. risticii* Minnesota strain, two of the Canadian strains clustered with the Kentucky (Herodia) and Maryland (Illinois) isolates, and three strains clustered with TW2-1, an *N. risticii* isolate from an *A. oregonense* adult gravid trematode isolated from the intestines of Eptesicus fuscus (bat) in Pennsylvania (1, 35) (Fig. 3).

10.1128/mBio.03429-19.1 FIG S1 P51 external loop 2 amino acid sequence alignment (A) and percent identity and divergence (B) of 3 strains of *Neorickettsia finleia*, 15 strains of *N. risticii*, 3 *N. sennetsu* strains, 2 SF agents, and 1 *Neorickettsia* strain from *F. hepatica.* Dashes, sequence not available or deletion; dots, identical to the majority of amino acids. Download FIG S1, PDF file, 0.9 MB. Copyright © 2020 Teymournejad et al. 2020 Teymournejad et al. This content is distributed under the terms of the Creative Commons Attribution 4.0 International license.

10.1128/mBio.03429-19.6 TABLE S1 Primers and sequences utilized for PCR amplification. Download Table S1, PDF file, 0.2 MB. Copyright © 2020 Teymournejad et al. 2020 Teymournejad et al. This content is distributed under the terms of the Creative Commons Attribution 4.0 International license.

**FIG 4:**
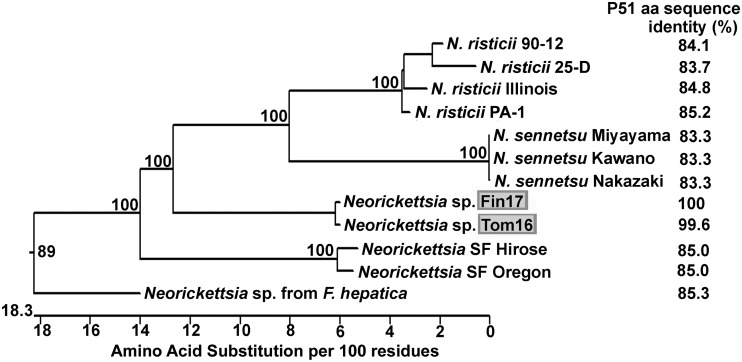
Phylogenetic tree of nearly full-length P51 amino acid sequences encoded in the genomes of these 12 *Neorickettsia* culture isolates. Positions of the deduced P51 sequences that are 470 residues long for *Neorickettsia* sp. Fin17 and Tom16 culture isolates (highlighted in gray) and those of closely related *Neorickettsia* strains are shown on the phylogenetic tree. Note the clustering of the Fin17 and Tom16 sequences, which are separated from the *N. risticii* clade. Bootstrap values (>50) for 1,000 replicates are shown at each branch point. Percent values to the right of the strain names indicate amino acid identity values obtained in comparison with strain Fin17^T^.

To verify that Fin17 and Tom16 are unique, almost full-length P51 DNA sequences were obtained to compare them with longer P51 sequences available in the GenBank database. The longest P51 amino acid sequences (253 residues, which could be aligned unambiguously) of three strains, Fin17, Tom16, and *Neorickettsia* sp. 081, were found to be identical (Fig. S2A). Comparison of even longer, almost full-length P51 sequences between residues 16 and 485 (the numbering is that of *N. risticii* Illinois P51 and includes deletions) that were available in the GenBank database revealed that the Fin17 and Tom16 P51 amino acid sequences were almost identical (99.6%; 460/462). However, they were distinct from the *N. risticii*, *N. sennetsu*, and SF agent sequences (Fig. 4 and Fig. S2A and B). In addition, the longer P51 sequence of *Neorickettsia* sp. from *F. hepatica* was distant from the phylogenetic clade of *N. risticii*, *N. sennetsu*, and the SF agents (Fig. 4). The *N. helminthoeca* P51 amino acid sequence was quite divergent from those of the other *Neorickettsia* strains (45) (Fig. S2B) and therefore was not included in the phylogenetic tree analysis, as we focused on P51 sequences of *Neorickettsia* spp. that are closely related to *N. risticii* and the newly isolated *Neorickettsia* sp. described here. The divergence (corrected levels of amino acid differences for common pairwise homologous sites for up to 462 amino acids [aa]) of P51 amino acid sequences among *N. risticii* isolates is 1.3 to 3.7 (Fig. S2B). The divergence of P51 amino acid sequences of *N. risticii* isolates from *N. sennetsu* isolates is 12.6 to 15.7 (Fig. S4). The divergence of P51 amino acid sequences of *N. risticii* isolates from Fin17 and Tom16 is 16.5 to 18.4. Thus, *N. risticii* is more divergent from Fin17 and Tom16 than from *N. sennetsu* (Fig. S2B), suggesting that Fin17 and Tom16 may belong to a new *Neorickettsia* species.

10.1128/mBio.03429-19.2 FIG S2 (A) Long P51 amino acid sequence alignment of three strains of *Neorickettsia finleia* and available long P51 amino acid sequences of other *Neorickettsia* species. (B) Percent identity and divergence of two strains of *Neorickettsia finleia* and available long P51 amino acid sequences of other *Neorickettsia* species. Download FIG S2, PDF file, 0.5 MB. Copyright © 2020 Teymournejad et al. 2020 Teymournejad et al. This content is distributed under the terms of the Creative Commons Attribution 4.0 International license.

### 16S rRNA gene sequence analysis.

**FIG 5:**
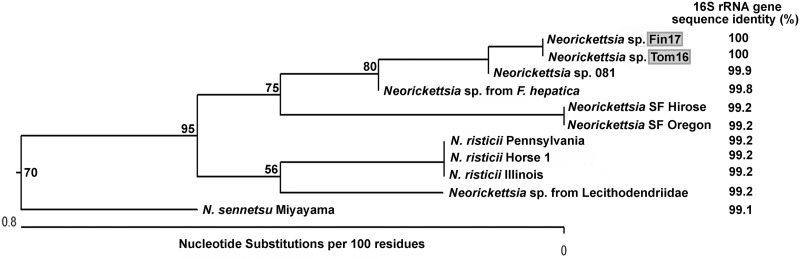
Phylogenetic tree of 16S rRNA gene sequences in the genomes of *Neorickettsia* culture isolates. The positions of the PCR-amplified 16S rRNA gene sequences (1,332 bp) from the *Neorickettsia* sp. Fin17 and Tom16 culture isolates (highlighted in gray) and those of closely related *Neorickettsia* strains are shown on the phylogenetic tree. Note the clustering of the *Neorickettsia* sp. Fin17, Tom16, and 081 sequences, which are separated from the *N. risticii* clade. Bootstrap values (>50) for 1,000 replicates are shown at each branch point. Percent values to the right of the strain names indicate nucleic acid identity values obtained in comparison with strain Fin17^T^.

We obtained almost full-length 16S rRNA gene sequences of Fin17 and Tom16. Using the longest 16S rRNA gene sequences (1,405 bp) that were unambiguously aligned, we found that those of Fin17 and Tom16 were identical, both of which differed from *Neorickettsia* sp. 081 at only 3 nucleotide positions (Fig. S3A). Using the longest 16S rRNA gene sequences (1,332 bp) that were available in the GenBank database for phylogenetic analysis, the 16S rRNA sequences of Fin17, Tom16, and *Neorickettsia* sp. 081 clustered near that of the *Neorickettsia* sp. from *F. hepatica* and were divergent from those of the SF agent strains (Fig. 5), unlike the P51 sequences (Fig. 3). These three clusters belonged to one clade, which was clearly separated from the cluster of *N. risticii* strains that included the 16S rRNA gene of *Neorickettsia* sp. from Deropegus aspina cercaria (family Lecithodendriidae) collected in Corvallis, OR (Fig. 5) (9). The Fin17/Tom16 and *Neorickettsia* sp. 081 16S rRNA gene sequences differed by 11 to 13 bp out of 1,405 bp relative to that of *N. risticii* Illinois, by 10 bp out of 1,332 bp relative to that of the SF agents, and by 2 to 5 bp out of 1,405 bp with that of the *Neorickettsia* sp. from *F. hepatica* (Fig. S3A). The divergence (corrected levels of nucleotide differences for common pairwise homologous sites for up to 1,332 bp) of 16S rRNA gene sequences among the three *N. risticii* isolates was zero (Fig. S3B). The divergence of 16S rRNA gene sequences of *N. risticii* isolates from *N. sennetsu* isolates was 0.8. The divergence of 16S rRNA gene sequences of *N. risticii* isolates from Fin17, Tom16, and *Neorickettsia* sp. 081 was 0.8. Thus, *N. risticii* divergence from Fin17, Tom16, and *Neorickettsia* sp. 081 was on par with that from *N. sennetsu* (Fig. S3B). Together, these data suggest that Fin17, Tom16, and *Neorickettsia* sp. 081 constitute a new species of *Neorickettsia*.

10.1128/mBio.03429-19.3 FIG S3 (A) 16S rRNA gene-based sequence alignment of three strains of *Neorickettsia finleia* and other *Neorickettsia* species. (B) Percent identity and divergence of two strains of *Neorickettsia finleia* and other *Neorickettsia* species. Download FIG S3, PDF file, 0.5 MB. Copyright © 2020 Teymournejad et al. 2020 Teymournejad et al. This content is distributed under the terms of the Creative Commons Attribution 4.0 International license.

### Strain-specific antigen 3 (Ssa3) sequence analysis.

**FIG 6:**
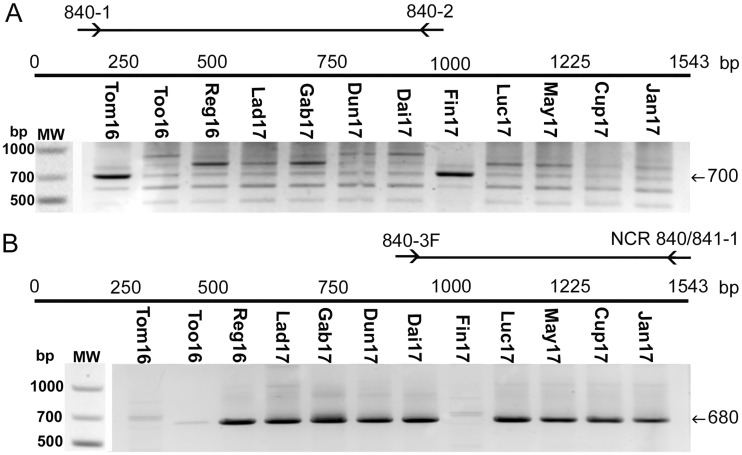
PCR amplification of the *Neorickettsia ssa3* nucleotide sequences from the 12 *Neorickettsia* culture isolates described here. DNAs from the culture isolates of the blood of the infected horses were subjected to PCR using the *Neorickettsia ssa3* sequence near their 5′ and 3′ conserved regions and the primers 840-1 and 840-2 (A) and 840-3F and NCR 840/841-1 (B), respectively. The amplified regions are depicted as horizontal bars flanked by the two facing arrows that delineate the positions of the two sets of primers. The expected PCR product sizes are 700 bp for the 5′ conserved region (A) and 680 bp for the 3′ conserved region (B). Note the multiple bands for *N. risticii* strains in panel A due to amplifications of various repeat regions but the primary 700-bp band for Tom16 and Fin17. The 680-bp band was not found in panel B for Tom16 and Fin17. MW, standard molecular size markers.

**FIG 7:**
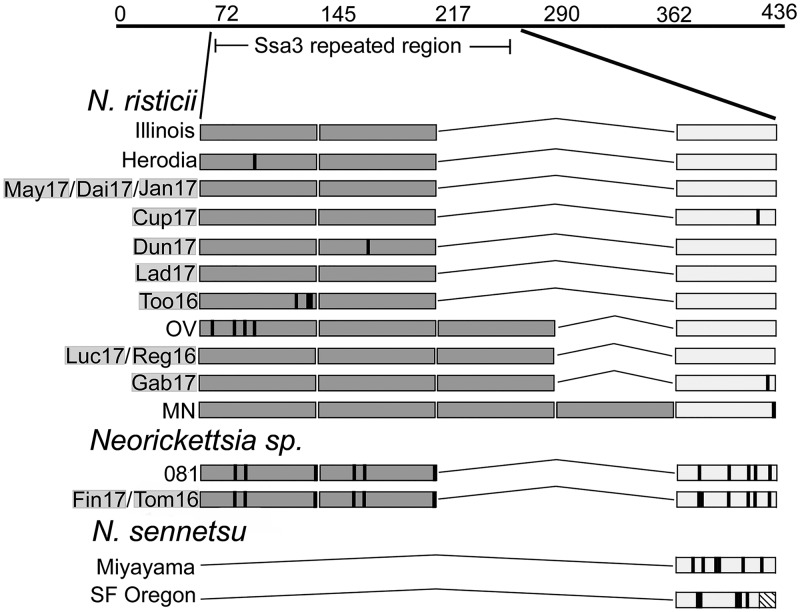
Repeat sequence patterns for the Ssa3 proteins from these 12 *Neorickettsia* isolates. The amplified Ssa3 repeat regions of the sequenced Ssa3s from these 12 isolates (highlighted in light gray) and various other *Neorickettsia* spp. aligned in relation to aa 53 to 196 of Ssa3 from *N. risticii* Illinois are shown. The medium gray boxes identify the positions of the 52-aa repeats. The open boxes identify the positions of the terminal 40-aa regions found for all *Neorickettsia* strains. The black lines indicate residue mutations in relation to the Ssa3 sequence of *N. risticii* Illinois. The box containing diagonal lines for SF agent Oregon delineates a 9-residue truncation in the terminal 40-aa region in relation to the other *Neorickettsia* species Ssa3 sequences. OV, one of Kentucky strains (29, 34); MN, Minnesota strain.

**FIG 8:**
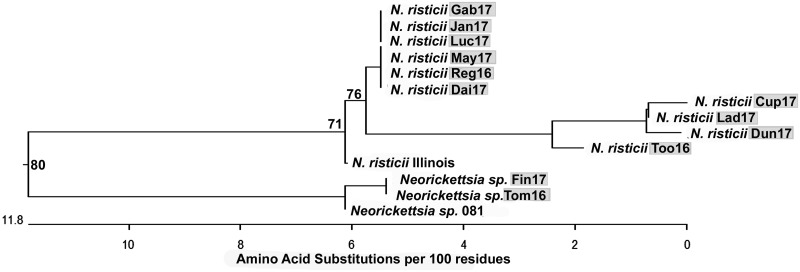
Phylogenetic tree of the adjacent two N-terminal 52-aa Ssa3 repeats from the 12 *Neorickettsia* isolates. The sequences of the PCR products from the Ssa3 N-terminal repeats (543 bp for *N. risticii ssa3*) shown in Fig. 6 and 7 were translated into their protein sequences (110 residues) and used to build a tree that included other closely related *Neorickettsia* Ssa3 sequences. Note the clustering of *Neorickettsia* sp. Fin17, Tom16, and 081 sequences, which are separated from the *N. risticii* clade. Bootstrap values (>50) for 1,000 replicates are shown at each branch point.

We PCR amplified the entire *ssa3* gene from the 12 *Neorickettsia* strains using three different sets of primer pairs that anneal to the conserved sequences within *ssa3* of *N. risticii* strains (Table S1). The primer pair 840-1/840-2 for the N-terminal half of Ssa3 (Ssa3N) produced multiple bands by single-step PCR in all strains due to the amplification of the various repeat regions. For Tom16 and Fin17, the primer pair amplified a primary product of 700 bp, whereas this band was much weaker in all 10 *N. risticii* strains (Fig. 6). Using the NCR-3F and NCR840/841-1 primers, heminested PCR allowed a band of 680 bp of the C-terminal half of Ssa3 to be amplified from the 10 *N. risticii* genomes; however, a band of the same mass was not amplified when the Tom16 and Fin17 genomes were used as the templates (Fig. 6). We sequenced the PCR-amplified *ssa3* products from 12 isolates to characterize their intramolecular repeats. We previously found that within the N-terminal halves of the Ssa3 protein repeat region of *N. risticii* strains, two to four tandemly arrayed 52-aa-residue repeats exist, whereas the Ssa3s of *N. sennetsu* strains and SF agents lack the repeats (34). *N. risticii*, *N. sennetsu*, and SF agent Ssa3s all have a terminal 40-aa repeat with a sequence similar to those of the 52-aa repeats (the above-mentioned sequences were 50% identical compared with the *N. risticii* Illinois repeat; E value of 6 × 10^−8^ based on protein-protein BLASTp analysis). When amino acid sequences were aligned according to the deduced *N. risticii* Illinois Ssa3 sequence between positions 72 and 250, it was found that the Fin17, Tom16, and 081 *Neorickettsia* species Ssa3s contain two tandem 52-aa repeats and a 40-aa terminal peptide (Fig. 7). Thus, in terms of the repeated sequences, those of the three *Neorickettsia* strains are similar to those of the *N. risticii* strains and distinct from those of the *N. sennetsu* strain and the SF agents. The Ssa3 sequences from the 10 *N. risticii* isolates have two or three 52-aa residue repeats (Fig. 7). Phylogenetic analysis of the amino acid sequences of the two N-terminal 52-aa repeats (the deduced sequences corresponding to amino acid positions 72 to 105 of *N. risticii* Illinois Ssa3) showed that those of Fin17, Tom16, and 081 were clustered and distinct from those of the *N. risticii* strains (Fig. 8 and Fig. S4).

10.1128/mBio.03429-19.4 FIG S4 Ssa3 N-terminal two-repeat amino acid sequence alignment of three strains of *Neorickettsia finleia* and other *Neorickettsia* species. Download FIG S4, PDF file, 0.2 MB. Copyright © 2020 Teymournejad et al. 2020 Teymournejad et al. This content is distributed under the terms of the Creative Commons Attribution 4.0 International license.

### Ssa1 sequence analysis.

**FIG 9:**
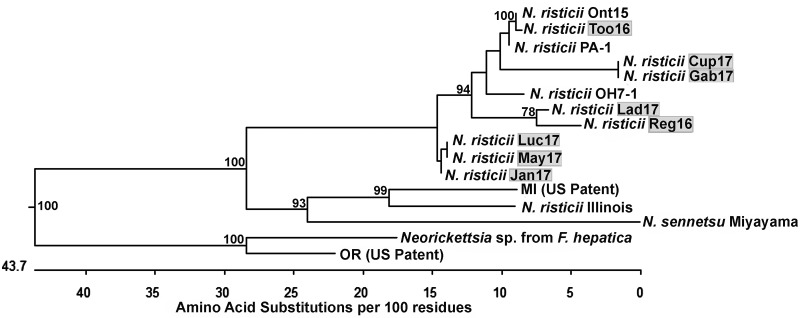
Phylogenetic tree of a 241-aa Ssa1 sequence from eight of the *Neorickettsia* isolates described in this study. DNA from the 12 *Neorickettsia* isolates was subjected to PCR for *Neorickettsia ssa1* using the primer set 838-1a/838-4 or Ssa1OT-R. PCR products corresponding to *ssa1* were detected for only eight isolates (highlighted in gray). The deduced amino acid sequences of these eight *ssa1* genes were aligned with closely related Ssa1 sequences from other *Neorickettsia* spp. Note the highly variable nature of Ssa1. Bootstrap values (>50) for 1,000 replicates are shown at each branch point. MI, Michigan strain; OR, oregon strain.

The Ssa1 protein has extensive intramolecular repeats (46). Using the 838-4/838-1a primer pair (Table S1), we amplified a nonrepeat region of *ssa1* (amino acid residues 301 and 473 of Ssa1 of *N. risticii* Illinois) for 8 of the *N. risticii* isolates but not for the new *Neorickettsia* sp. (Fin17 or Tom16) or the *N. risticii* isolates (Dai17 and Dun17). Phylogenetic analysis showed that the 8 deduced aa sequences clustered with known Ssa1 sequences from *N. risticii* strains isolated in the eastern and Midwestern regions of the United States (Fig. 9 and Fig. S5). Newly obtained sequences used in the analysis shown in Fig. 2 to 5 and Fig. 7 to 9 and their GenBank accession numbers are summarized in Table 2.

**TABLE 2:** *Neorickettsia* sequences amplified and compared

Sample	Fragment size obtained (bp)	Fragment size compared (bp)	Gene amplified	GenBank accession no. (DNA)
*N. finleia* sp. nov. Fin17	1,386	1,386	*p51*	MH428610
	716	513	*ssa3*	MH814541
	1,405	1,323	16S rRNA	MH476282

*N. finleia* sp. nov. Tom16	1,386	1,386	*p51*	MH428617
	714	513	*ssa3*	MH814542
	1,405	1,323	16S rRNA	MH476283

*N. risticii* Lad17	259	259	*p51*	MH428614
	586	450	*ssa1*	MH428620
	873	474	*ssa3*	MH814750

*N. risticii* Gab17	262	262	*p51*	MH428613
	478	342	*ssa1*	MH428623
	1,461	513	*ssa3*	MH814749

*N. risticii* Dun17	259	259	*p51*	MH428612
	714	474	*ssa3*	MH814748

*N. risticii* Dai17	259	259	*p51*	MH428611
	1,251	513	*ssa3*	MH814747

*N. risticii* Luc17	262	262	*p51*	MH428609
	656	519	*ssa1*	MH428619
	1,476	513	*ssa3*	MH814746
				
*N. risticii* May17	262	262	*p51*	MH428608
	656	519	*ssa1*	MH428618
	1,288	513	*ssa3*	MH814744

*N. risticii* Cub17	262	262	*p51*	MH428607
	478	342	*ssa1*	MH428622
	766	474	*ssa3*	MH814745

*N. risticii* Jan17	259	259	*p51*	MH428606
	656	507	*ssa1*	MH428625
	1,080	513	*ssa3*	MH814743

*N. risticii* Too16	259	259	*p51*	MH428616
	587	450	*ssa1*	MH428624
	987	513	*ssa3*	MH814752

*N. risticii* Reg16	269	269	*p51*	MH428615
	524	390	*ssa1*	MH428621
	1,132	513	*ssa3*	MH814751

10.1128/mBio.03429-19.5 FIG S5 Ssa1 amino acid sequence alignment of *Neorickettsia* species. Download FIG S5, PDF file, 0.7 MB. Copyright © 2020 Teymournejad et al. 2020 Teymournejad et al. This content is distributed under the terms of the Creative Commons Attribution 4.0 International license.

### Experimental infection of naive ponies.

**FIG 10:**
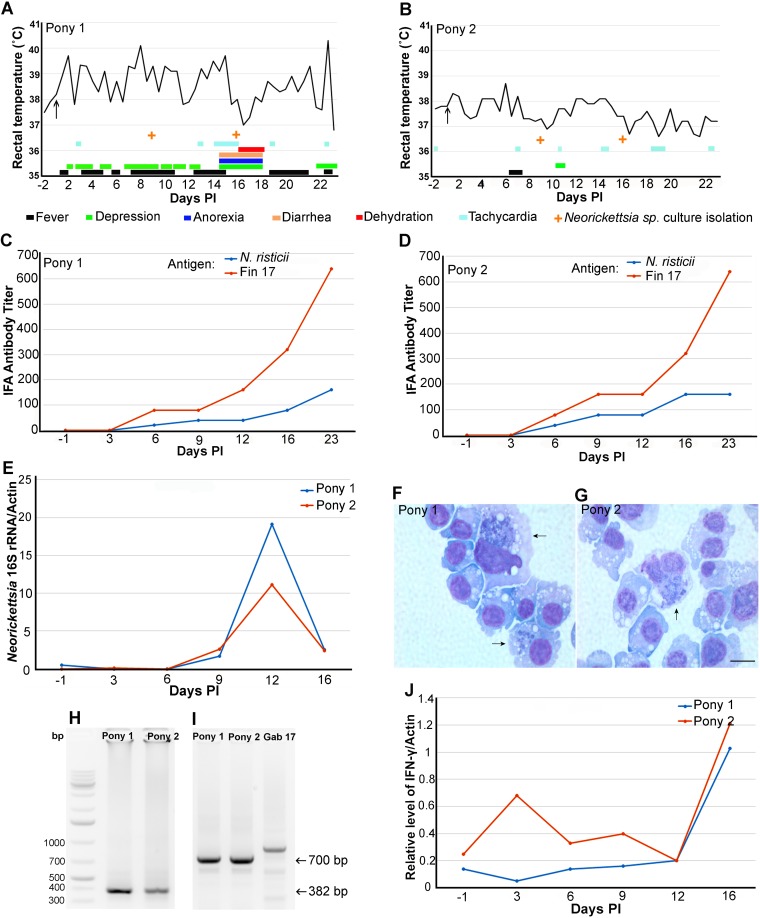
Experimental inoculation of ponies with Fin17. (A and B) Clinical signs in ponies 1 and 2 following Fin17 inoculation. A rectal temperature of >38.7°C is recorded as fever (the normal adult equine rectal temperature is 37.5°C to 38.6°C). A heart rate of >50 beats per min is recorded as tachycardia (the normal heart rate of horses is 20 to 50 beats per min). Depression, anorexia, diarrhea, and dehydration were recorded. Diarrhea of pony 1 is cow pie to pipe stream. PI, postinoculation. (C and D) IFA development using *N. risticii* Pennsylvania and Fin17 as antigens. (E) Relative neorickettsemia (*Neorickettsia* 16S rRNA gene normalized by equine actin) of ponies 1 and 2 during the course of infection as determined by PCR. (F and G) Fin17 culture isolates from the blood of both ponies at day 9 postinoculation. Diff-Quick staining is shown. Arrows point to the infected cells. Bar, 10 μm. (H and I) PCR of DNAs extracted from the culture isolates from ponies at day 9 postinoculation. Nested PCR detecting *Neorickettsia* sp. 16S rRNA (31) (382 bp) (H) and Fin17 *ssa3N* (700 bp) (I), respectively, were performed. Gab17 was used as an *ssa3N N. risticii* control. (J) IFN-γ, interferon gamma responses normalized by equine actin of ponies 1 and 2 during the course of infection as determined by RT-PCR.

Ponies 1 and 2 were intravenously inoculated with Fin17-infected P388D_1_ cells. Clinical signs in pony 1 were continuous bouts of fever and depression, and intermittent tachycardia and anorexia and watery diarrhea on days 14 to 18. Dehydration and a hematocrit increase followed the diarrhea (Fig. 10A). Pony 2 developed only a slight fever on day 7 and depression on day 11 (Fig. 10B). Both ponies seroconverted at day 6: indirect fluorescent-antibody assay (IFA) titers for both ponies using Fin17 as the antigen were much higher than those using *N. risticii* PA-1 as the antigen throughout the study (Fig. 10C and D). *Neorickettsia* sp. was detected by quantitative PCR (qPCR) in the peripheral blood specimens from both ponies starting at day 9 postinoculation and peaked at day 12 (Fig. 10E). Culture isolations were attempted for blood specimens from both ponies on days 9 and 16, and all cultures were positive, indicating live bacteria circulating in the blood: (i) by Diff-Quik staining, dark blue to purple cocci were found in both morulae (microcolonies) and individual forms that tended to occupy one side of the cytoplasm of P388D_1_ cells (Fig. 10F and G); (ii) by PCR using primers specific for 16S rRNA and *ssa3N*, specific amplified products of Fin17 organisms from ponies 1 and 2 were detected (Fig. 10H and I); and (iii) sequencing of the PCR product of *ssa3N* showed that the culture isolates from both ponies were identical to each other and to the original Fin17 horse isolate. These data fulfill Koch’s postulates that Fin17 is the causative agent of PHF.

Interferon gamma has a critical role in inhibiting *N. risticii* infection of macrophages in culture (47). Thus, the interferon gamma mRNA levels in peripheral blood leukocytes were determined by reverse transcription-quantitative PCR (qRT-PCR), following the time course of infection. Pony 2 rapidly upregulated interferon gamma expression upon Fin17 inoculation, whereas pony 1 was unable to induce a rapid interferon gamma response (Fig. 10J). Nevertheless, both ponies eventually induced higher levels of interferon gamma expression at day 16 postinoculation (Fig. 10J), which coincided with the reduction of bacterial loads in the peripheral blood (Fig. 10E).

### Whole-genome sequencing and comparison among *Neorickettsia* spp.

**FIG 11:**
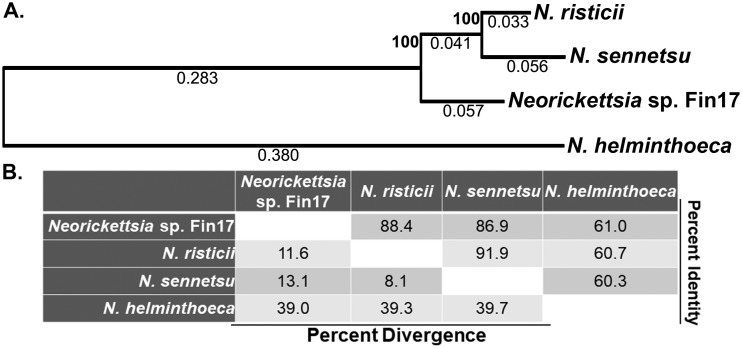
Phylogenetic tree of concatenated proteins among *Neorickettsia* spp. (A) Seven protein sequences (Eno/GltA/GroEL/DsbB/P51/Nsp1/Ssa3) from the four *Neorickettsia* spp. were aligned individually using MegAlign. The aligned sequences were concatenated (2,804 aa total) and converted to PHYLIP format. Bootstrap values for 1,000 replicates were obtained using PHYLIP with the neighbor-joining method. The tree was drawn to scale, with branch lengths (average nucleotide substitutions per site) shown under each branch and bootstrap values shown at each branching point. (B) Percent identity and divergence of amino acid sequences of 7 concatenated proteins of *Neorickettsia finleia* and three other *Neorickettsia* spp. Phylogenetic tree and evolutionary distance analyses were performed with CLUSTAL MUSCLE in the MegAlign Pro program (DNAStar).

**FIG 12:**
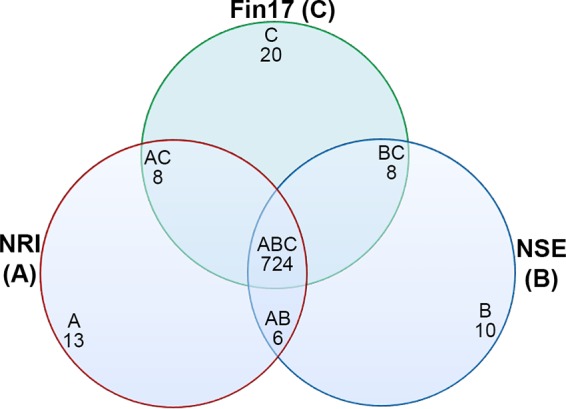
Numbers of protein orthologs shared among *Neorickettsia* spp. A Venn diagram was constructed to demonstrate the numbers of conserved and unique genes between *Neorickettsia* spp., which were determined by reciprocal BLASTp using an E value of <e^−10^. Numbers within the intersections of different circles indicate ortholog clusters conserved within 2 or 3 organisms. NRI, *N. risticii* (A); NSE, *N. sennetsu* (B); Fin17, *Neorickettsia* sp. Fin17 (C).

To better understand the new *Neorickettsia* species, we determined the complete genome sequence of the Fin17 strain. Fin17 has a single double-stranded circular chromosome of 864,092 bp with an overall GC content of 41%, similar to those of all other genome-sequenced *Neorickettsia* spp. (*N. risticii*, *N. sennetsu*, and *N. helminthoeca*) (45, 46, 48) (Table 3). In addition to P51 and Ssa proteins described above, Fin17 contained three tandem *Neorickettsia* surface proteins (Nsps), which are found in all *Neorickettsia* spp. sequenced so far (Table S3). To more accurately estimate the evolutionary distance of *Neorickettsia* sp. Fin17 from other *Neorickettsia* spp., phylogenetic analysis was performed using the concatenated protein sequences (2,804 aa total) of four conserved housekeeping genes (Eno/GltA/GroEL/DsbB) and three surface proteins (P51/Nsp1/Ssa3). By this analysis, *N. risticii* is more divergent from Fin17 than from *N. sennetsu* (Fig. 11), in agreement with the phylogenetic analysis of individual proteins or genes (P51, Ssa3, and 16S rRNA) (Fig. 2 to 9). To compare the genomic contents among *Neorickettsia* sp. Fin17 and two closely related spp., *N. risticii* and *N. sennetsu*, 2- and 3-way comparisons were performed using reciprocal BLASTp. The numbers of shared or unique proteins were plotted in a Venn diagram, and 3-way comparison results showed that the majority of proteins are conserved among the three *Neorickettsia* spp. (Fig. 12). Two-way comparisons revealed that Fin17 had more unique proteins that were not shared with *N. risticii* or *N. sennetsu*, indicating that Fin17 belongs to a new *Neorickettsia* sp.

**TABLE 3:** Genome properties of representative *Neorickettsia* species

Parameter	Value for *Neorickettsia* species^*a*^			
	Fin17	NRI	NSE	NHO
GenBank accession no.	NZ_CP047224	NC_013009.1	NC_007798.1	NZ_CP007481.1
Size (bp)	864,092	879,977	859,006	884,232
GC content (%)	40.7	41.3	41.1	41.7
No. of proteins	735	754	760	788
No. of tRNAs	33	33	33	33
No. of rRNAs	3	3	3	3
No. of other RNAs	3	3	3	3
No. of pseudogenes	17	15	2	14
Total no. of genes	791	807	801	841

a Abbreviations: Fin17, *Neorickettsia* sp. Fin17 (data obtained in this study); NRI, *N. risticii* Illinois (46); NSE, *N. sennetsu* Miyayama (48); NHO, *N. helminthoeca* Oregon (45).

## DISCUSSION

For this study, we culture isolated 12 *Neorickettsia* strains from the blood of horses with typical PHF clinical signs that resided at various locations in Ontario, Canada, during 2016 and 2017. As both blood and fecal samples were real-time *N. risticii* PCR (41) negative for some horses, the 12 culture isolates were analyzed by PCR of four genes, P51, 16S rRNA, Ssa3, and Ssa1, followed by sequencing. Phylogenetic analysis revealed that only 10 of the isolates were *N. risticii* strains, whereas the remaining two isolates (Fin17 and Tom16) were a previously uncharacterized *Neorickettsia* sp. The results were corroborated by whole-genome sequencing of Fin17 and genomic comparison with *N. risticii*, *N. sennetsu*, and *N. helminthoeca* (21, 45, 46, 48). Experimental inoculation of Fin17 clearly showed that it can infect horses and cause PHF or subclinical infection. Subclinical infection with *Neorickettsia* is not unusual. Our previous epidemiological studies of a total of 1,400 horses at two race tracks in Ohio in 1986 revealed that 13 and 20% of horses, respectively, on the ground had IFA titers of 20 to 10,240, and most of the seropositive horses lacked clinical signs (49), suggesting subclinical infection with *N. risticii* or infection with serologically cross-reactive *Neorickettsia* species.

Because surface-exposed PHF-related proteins, such as P51 and the Ssa proteins, are linked to strain-dependent antigenic variation, they are potential candidates for diagnosis and vaccine development. Therefore, for this report, we initially focused on P51 and Ssa sequences when assessing strain variations. Native P51 of *N. sennetsu* has porin activity, which is an important means for nutrient acquisition among Gram-negative bacteria (50). The *p51* gene is found in *N. risticii*, *N. sennetsu*, *N. helminthoeca*, the SF agent, and *Neorickettsia* species from *F. hepatica* (21, 45, 46, 48). P51 is the major surface-exposed protein of *N. risticii* strains and is highly immunogenic in animals infected with *N. risticii* or *N. helminthoeca* (21, 34, 45, 51). The bacterial Ssa protein was initially thought to be encoded by a single gene and may be an immunologically reactive surface protein that could be developed as a vaccine (32, 52, 53). Because a signal peptide sequence is not predicted for Ssa3 (46), if it is a bacterial surface protein, it is likely to be secreted in a Sec-independent manner (32, 52, 53). Our previous whole-genome sequence analyses of *N. risticii* and *N. sennetsu* revealed that they harbored three and two (*ssa2* has degenerated in *N. sennetsu*) tandemly repeated *ssa* genes, respectively (46, 48), whereas *N. helminthoeca* carries only a single *ssa* gene (45). The current genome analysis showed that Fin17 carries 3 *ssa* genes (see Table S3 in the supplemental material). Interestingly, unlike tandem *ssa* genes in the same direction in *N. risticii* and *N. sennetsu*, the *ssa3* gene of Fin17 is in the opposite direction downstream of the *ssa1-ssa2* gene cluster. These findings suggest that the multiple *ssa* genes in Fin17, *N. risticii*, and *N. sennetsu* arose as gene duplication events after the two species diverged from *N. helminthoeca.* Ssas have extensive intramolecular sequence repeats (34, 45, 46, 48).

Notably, the Fin17/Tom16 16S rRNA and P51 gene sequences were phylogenetically separated from those of other PHF-related strains, whereas the presence of Ssa3 repeat structures was similar to those of the *N. risticii* strains and distinct from those of *N. sennetsu* and the SF agents, suggesting that Ssa3 may be involved in PHF infection and pathogenesis. As the *ssa3* primers used here can amplify both *N. risticii* and the new *Neorickettsia* species, PCR amplification and amplicon size comparison of *ssa3* may be used to discern new *Neorickettsia* spp. from more commonly recognized *N. risticii* strains. In agreement with a previous study (34), the Ssa1 sequences from *N. risticii* strains are highly variable or were undetectable by using primers designed based on the conserved region and thus may not be suitable as the target for diagnosis.

rRNAs are considered to be the ultimate molecular chronometers (54), because (i) they are found in all bacterial species, (ii) they are required for proper ribosomal structure and function, and (iii) their sequence variation follows passive molecular evolution because they are not under selective pressure induced by the environment, e.g., as it occurs in bacterial surface proteins in response to host immune systems. Among the various types of rRNAs, 16S rRNA gene sequences are easily obtained and are sufficient in length for reliable comparisons; they also represent the greatest amount of data in GenBank. The average substitution rate for 16S rRNAs in eubacteria is estimated to be 1% per 50 million years (55, 56), which suggests that the Fin17, Tom16, and *Neorickettsia* sp. 081 16S rRNAs may have diverged from those of *N. risticii*, the SF agents, and the *Neorickettsia* sp. from *F. hepatica* between 39 million and 46 million years ago, 38 million years ago, and between 11 million and 18 million years ago, respectively. Because *N. risticii* 16S rRNA gene-based PCR is widely used for the clinical diagnosis of PHF, primers for this new *Neorickettsia* sp. need to be developed to avoid false-negative results when assessing the status of PHF-related agents from the field, as happened here for Fin17 and Tom16. Examples of effective PCR templates and primers that can be used for the detection of known PHF agents are reported here.

If therapeutic intervention does not occur early, the course of PHF is usually 5 to 10 days, with the mortality rate being 17 to 36%. Owing to the short diagnostic window and dire emotional and economic impacts on horse owners and the equine industry, an effective PHF vaccine is much needed. Currently, the inactivated whole-cell vaccines that are commercially available provide only limited protection (33). This lack of protection was clearly demonstrated in that the two horses examined in this study that had been vaccinated developed typical signs of PHF, and upon culture, live *N. risticii* and the new *Neorickettsia* isolates were obtained from their blood. How prevalent the new *Neorickettsia* sp. is in nature is not known; however, our results suggest that in Ontario, Canada, a substantial proportion of horses, 2/12 (16.6%), were included with this new species. Including this new species in a newly designed PHF vaccine could improve vaccine efficacy.

As a result of its long endosymbiotic lifestyle in trematode hosts, the genome sizes of *Neorickettsia* spp. are only 0.86 to 0.88 Mb, and ∼60% of their genomes encode housekeeping proteins (45, 48, 57). As obligatory intracellular bacteria of trematodes, *Neorickettsia* spp. have little opportunity to interact with other bacteria that could increase their genetic variation by gene transfer. Nonetheless, the *ssa* sequences of *Neorickettsia* spp. are highly strain dependent (29, 34, 43, 44). *Neorickettsia* may be able to generate genetic variation owing to a limited set of proteins that can repair gene mutations (45, 48, 57).

The occurrence of PHF is associated with the presence of a specific type of freshwater snail, which is the first intermediate host of trematodes that harbor *Neorickettsia* (42, 58). Although snails or trematodes associated with PHF had previously not been reported in Canada, most PHF cases that have been diagnosed there have been found near freshwater, e.g., near Lake St. Clair, Lake Ontario, Lake Erie, and Lake Simcoe (26), including the first case of PHF in Canada for which an *N. risticii* strain (Ont15) was culture isolated (Fig. 1). The genomic sequence of Ont15 is distinct from those of other sequenced *N. risticii* strains, although the strain belongs to the Midwestern ecotype (30). Each type of *Neorickettsia* sp. seems to infect a specific trematode species, which in turn parasitizes one of three specific hosts at each trematode developmental stage, i.e., the primary intermediate host (gastropods), the secondary intermediate host, and the definitive host (1, 7–9, 35). Sporocysts or rediae develop into cercariae in the primary intermediate hosts and encyst as metacercariae in secondary intermediate hosts, and mature adult gravid trematodes develop in the gut lumen of definitive hosts (7). The *Neorickettsia* sp. from *F. hepatica*, which encysts in aquatic plants (59), is closely related to PHF agents. The effects of *Neorickettsia* species in *F. hepatica* on humans, cattle, or horses remain to be elucidated. To do so, culture isolates of that *Neorickettsia* sp. are needed. The trematode host(s) of the new Canadian *Neorickettsia* sp. and the life cycle of its encysted trematode remain to be studied. The results reported here should help find the natural reservoirs of this *Neorickettsia* sp.

Because *Neorickettsia* spp. are obligatory intracellular bacteria, their survival, distribution, and population density are dependent on the host trematode population. In turn, the survival and population density of the trematode (the obligatory endoparasite) are dependent on their two intermediate and definitive hosts, of which mollusks are the first and essential intermediate hosts. Because of this double parasitic relationship, *Neorickettsia* spp. are expected to be very sensitive to changes in their natural environments, which include both pollution and climate effects. Consequently, the emergence of a *Neorickettsia* sp. population and its associated disease state as found in this study may reflect environmental changes.

This newly isolated Canadian *Neorickettsia* sp. strain is closely related to *Neorickettsia* sp. 081 isolated ∼26 years ago in Findley, OH. Given the molecular evidence, we suggest that this new species should be named Neorickettsia finleia, because it was first found in Findley, OH, the birthplace of Howard Ricketts, the discoverer of Rickettsia rickettsii. However, the type strain will be Fin17, as 081 was recently lost due to accidental liquid nitrogen dry-out.

### Description of “*Candidatus* Neorickettsia finleia” sp. nov.

*Neorickettsia finleia* (N.L. fem. adj. *finleia*, from Findley; the type strain, Fin17, was isolated from a horse from Ontario, Canada, in 2017).

To date, all infected horses have originated from Findley, OH, and Ontario, Canada. Horse infection with N. finleia causes an illness characterized by fever, anorexia, depression, and diarrhea. *N. finleia* and *N. risticii* are serologically cross-reactive. *N. finleia* grows well in the P388D_1_ murine macrophage cell line. The type strain, Fin17^T^, is available through the Centers for Disease Control and Prevention Rickettsial Isolate Reference Collection (CRIRC number NFI001^T^) and through the Collection de Souches de I’Unite des Rickettsies (CSUR deposit identification number Q1925).

## MATERIALS AND METHODS

### Ethics statement.

All animal experiments were performed in accordance with The Ohio State University Institutional Animal Care and Use Committee guidelines and approved e-protocol number 2008A0066. The University program has full continued accreditation by the Association for Assessment and Accreditation of Laboratory Animal Care International (AAALAC), number 000028, dated 18 April 1966, and has Public Health Service assurance renewal number A3261-01, dated 7 March 2019 through 28 February 2023. The program is licensed by the USDA, under license number 31-R-014, and is in full compliance with animal welfare regulations.

### PHF cases.

Twelve horses clinically diagnosed with PHF between 12 July and 16 September 2016 and between 26 June and 17 August 2017 were assessed. Horses resided near Lake St. Clair, Lake Erie, Lake Ontario, and/or Lake Simcoe in Ontario, Canada (Fig. 1). The number of days that the horse was observed to be sick by the owner before the attending veterinarian first examined the horse and collected blood samples, clinical signs, and the vaccination status of the horse are shown in Table 1. The last two numbers in each horse identifier indicate the year of *Neorickettsia* species isolation (e.g., Fin17 was isolated in 2017). The following clinical signs were recorded: depression, anorexia, rectal temperature, heart rates, color of mucous membranes (buccal and conjunctival), dehydration, gastrointestinal sounds and colic signs, nature of diarrhea (mild [softer than normal], moderate [“cow pie”], or severe [watery, profuse, or projectile] diarrhea), and laminitis (hoof pain).

Blood and fecal samples were tested by real-time PCR, which detects 85 bp of the 16S rRNA gene of *N. risticii*, using primers ER.133f (5-GTTATTCCCTACTACCAGGCAAGTTC-3′) and ER.54r (5′-AACGGAATCAGGGCTGCTT-3′), which amplified an 85-bp fragment of the 16S rRNA gene (41), at the Ontario Veterinary College Veterinary Teaching Hospital (OVC-VTH). Other common enteropathogens (such as *Salmonella* spp. and Clostridium difficile, etc.) causing diarrhea in horses were excluded.

### Culture isolation of *Neorickettsia*.

Approximately 50 ml of EDTA blood was collected from each horse and arrived within 48 h at the Rikihisa laboratory, The Ohio State University (Columbus, OH), for culture. Blood from each horse was centrifuged at 500 × *g* for 10 min to obtain the buffy coat. By lysing the remaining red blood cells in the buffy coat with an ammonium chloride solution, peripheral blood leukocytes were obtained (29). The leukocyte preparations were individually inoculated into P388D_1_ cell (American Type Culture Collection, Rockville, MD) preparations (29), which were then cultured in RPMI 1640 medium (Gibco, Grand Island, NY) containing 5% fetal bovine serum (FBS; Atlanta Biologicals, Norcross, GA) and 2 mM l-glutamine (Gibco) (31). Samples of the cultured cells were examined weekly for signs of infection under a light microscope after Diff-Quik staining (29). When infection was seen, ∼0.5 ml of the culture was harvested for the isolation of DNA, which was then used in a nested PCR test with *N. risticii*-specific primers for the bacterial 16S RNA gene to amplify a 382-bp sequence (31) (see Table S1 in the supplemental material). At least 75% of each culture medium was replaced with fresh RPMI 1640 medium containing 5% FBS and 2 mM l-glutamine weekly until infection was seen or the experiment ended by freezing infected cells.

### PCR, sequencing, and sequence analyses.

DNA was purified from the buffy coats of the PHF-positive horses or from *N. risticii* in the P388D_1_ cell cultures using DNeasy blood and tissue kit reagents (Qiagen, Valencia, CA). PCR amplification was performed using *Taq* DNA polymerase (New England BioLabs, Ipswich, MA), extracted genomic DNA as the template, and primers designed for conserved regions identified by alignment of multiple *Neorickettsia* strains and/or *N. risticii* strain DNA sequences of proteins (Table S1). The 16S rRNA gene, *p51*, *ssa1*, and *ssa3* were amplified using the primer pairs shown in Table S1. The PCR products were sequenced at The Ohio State University Comprehensive Cancer Center Nucleic Acid Shared Resource Facility. 16S rRNA gene sequences and deduced amino acid sequences of *p51*, *ssa1*, and *ssa3* were aligned, and phylogenetic and evolutionary distance analyses were performed with CLUSTAL W (60) in the MegAlign program of DNAStar (Madison, WI) Lasergene 12. To estimate confidence levels, bootstrap values for 1,000 replicates were obtained using PHYLIP, version 3.695 (with the programs SeqBoot, Protdist, Neighbor, and Consense) (61, 62). Homologous gene and protein sequences were found using the Basic Local Alignment Search Tool (BLAST) algorithms BLASTn and protein-protein BLASTp, respectively (63, 64).

### Experimental infection of ponies.

Two healthy female ponies, 90 to 100 kg, aged 5 years old, and negative by an *N. risticii* 16S rRNA nested PCR and by an indirect fluorescent-antibody assay (IFA), as previously described (31), were used. Ponies were intravenously inoculated with 3 × 10^7^ Fin17-infected P388D_1_ cells in 5 ml RPMI 1640 medium. Fever, depression, anorexia, diarrhea, leukopenia, laminitis, and other clinical signs were monitored daily. Blood samples were aseptically taken from the jugular vein at 2- to 4-day intervals to be used for IFA titer determination using *N. risticii* Pennsylvania and Fin17 as the antigens, buffy coat collection, PCR and RT-PCRs, complete blood counts, and chemical profiles. The study was terminated on day 23.

### Fin17 reisolation.

Citrated blood samples (∼8 ml) were centrifuged at 1,500 × *g* for 5 min. The plasma was removed and used to determine IFA titers. Buffy coats containing the mononuclear cell fraction were overlaid onto 5 ml of Histopaque 1077 (Sigma, St. Louis, MO) and centrifuged at 1,500 × *g* for 25 min. The interface containing mononuclear cells between Histopaque 1077 and remaining plasma was collected and resuspended in 1 ml of RPMI 1640 medium. The mononuclear cell suspension was added to a well of a 6-well plate containing a monolayer of P388D_1_ cells in 1 ml of RPMI 1640 medium containing 5% heat-inactivated FBS and 2 mM l-glutamine. Floating neutrophils and lymphocytes were removed after 2 days of culturing. Cultured cell samples were taken and examined every 3 to 4 days after centrifugation by using a Cytospin 3 centrifuge (Shandon, Inc., Pittsburgh, PA) and Diff-Quik staining (Baxter Scientific Products, Obetz, OH). For positive cultures, the number of days required for infection of more than 3% of P388D_1_ cells with more than 5 to 10 clearly identifiable organisms or a morula (inclusion) was recorded. The culture was considered negative when *Neorickettsia* was not found for >29 days of culture.

### IFA titration.

IFA titers were determined using *N. risticii* PA-1 strain- and *Neorickettsia* Fin17-infected P388D_1_ cells as the antigens, as previously described (31). Positive Alexa Fluor 488-anti-horse IgG (Jackson ImmunoResearch, West Grove, PA) staining of intracellular *Neorickettsia* at serum dilutions of >1:20 is considered a positive result (23).

### Reverse transcription-quantitative PCR.

The buffy coat was obtained from 10 ml blood, and red blood cells were lysed using ACK lysing buffer (Thermo Fisher). The cells were washed three times with RPMI 1640 medium, and total RNA was extracted using an RNeasy kit (Qiagen, Valencia, CA). RNA concentrations and quality were determined by using a NanoDrop spectrophotometer (Thermo Fisher). Total RNA (1 μg) was reverse transcribed using a Maxima H-Minus first-strand cDNA synthesis kit and random hexamers (Thermo Fisher). The qPCR mixture (25 μl) included 2 μl cDNA (corresponding to 0.2 to 0.3 μg of total RNA), 0.25 μM each primer, and 12.5 μl SYBR green qPCR master mix (Thermo Fisher). PCR was performed in an Mx3000P instrument (Stratagene) using equine actin and equine interferon gamma primers (65).

### Whole-genome sequencing and analysis.

Host-cell-free Fin17 was purified from 13 T175 flasks (13 × 10^8^ cells) of infected P388D_1_ cells as previously described, with slight modifications (45). Briefly, to improve the yield of Fin17 bacteria, infected cells were first Dounce homogenized 40 times, and the pellets were then resuspended in 1× SPK buffer (0.2 M sucrose and 0.05 M potassium phosphate [pH 7.4]) and sonicated at setting 3, 8 s each, 3 times with a W380 sonicator (Heat Systems, Newtown, CT). After stepwise centrifugation and filtration to remove unbroken cells and host nuclei, host-cell-free Fin17 bacteria were pelleted by centrifugation at 10,000 × *g* for 10 min. DNA was purified by using Genomic-tip 20/G (catalog number 10223; Qiagen) with a genomic DNA buffer set (catalog number 19060; Qiagen). Whole-genome sequencing, assembly, genome component analysis, gene function annotation, and circular genome data visualization were performed by Novogene Corporation, Inc. (Sacramento, CA). The coding genes, including the protein-coding open reading frames (ORFs), rRNAs, tRNAs, and small RNA (sRNA), were predicted by the NCBI Prokaryotic Annotation Pipeline (PGAP).

To calculate the numbers of conserved and unique proteins among *Neorickettsia* spp., protein ortholog clusters were determined by using reciprocal BLASTp with cutoff scores of an E value of <e^−10^. For phylogenetic analysis, 7 concatenated proteins, including 4 conserved housekeeping proteins (phosphopyruvate hydratase/enolase, citrate synthase [GltA], 60-kDa chaperone [GroEL], and disulfide bond formation protein [DsbB]) and 3 divergent outer membranes proteins (P51/Nsp1/Ssa3) from the four *Neorickettsia* spp. with completed genome sequences, were aligned individually using MegAlign. The aligned sequences were concatenated (2,804 aa total) and converted to PHYLIP format using the EMBOSS seqret program (https://www.ebi.ac.uk/Tools/sfc/emboss_seqret/). Phylogenetic tree and evolutionary distance analyses of the concatenated proteins were performed with CLUSTAL MUSCLE in the MegAlign Pro program (DNAStar). Bootstrap values for 1,000 replicates were obtained using PHYLIP as described above.

### Data availability.

GenBank accession numbers for the P51, Ssa3, and Ssa1 sequences of the Canadian *N. risticii* strains and the new *Neorickettsia* strain isolated in this study are shown in Table 2. The P51, Ssa3, and Ssa1 sequences used for the phylogenic analysis in this study are included in Table S2. The completed genome sequence of *N. finleia* Fin17 was deposited in GenBank (GenBank accession number NZ_CP047224).

10.1128/mBio.03429-19.7 TABLE S2 *Neorickettsia* sequences obtained from GenBank and used for phylogenetic analysis. Download Table S2, PDF file, 0.1 MB. Copyright © 2020 Teymournejad et al. 2020 Teymournejad et al. This content is distributed under the terms of the Creative Commons Attribution 4.0 International license.

10.1128/mBio.03429-19.8 TABLE S3 Putative outer membrane proteins of *Neorickettsia* spp. Download Table S3, DOCX file, 0.02 MB. Copyright © 2020 Teymournejad et al. 2020 Teymournejad et al. This content is distributed under the terms of the Creative Commons Attribution 4.0 International license.
