# Erratum for Teymournejad et al., “Isolation and Molecular Analysis of a Novel *Neorickettsia* Species That Causes Potomac Horse Fever”

**DOI:** 10.1128/mBio.00774-20

**Published:** 2020-04-21

**Authors:** Omid Teymournejad, Mingqun Lin, Hannah Bekebrede, Ahmed Kamr, Ramiro E. Toribio, Luis G. Arroyo, John D. Baird, Yasuko Rikihisa

**Affiliations:** a Laboratory of Molecular, Cellular, and Environmental Rickettsiology, Department of Veterinary Biosciences, College of Veterinary Medicine, The Ohio State University, Columbus, Ohio, USA; b Department of Veterinary Clinical Sciences, College of Veterinary Medicine, The Ohio State University, Columbus, Ohio, USA; c Department of Clinical Studies, Ontario Veterinary College, University of Guelph, Guelph, Ontario, Canada

## ERRATUM

Volume 11, no. 1, e03429-19, 2019, https://doi.org/10.1128/mBio.03429-19. In Discussion, “Findley, Ohio” should be “Findlay, Ohio,” and throughout the paper, “*Neorickettsia finleia* sp. nov.” should be “*Neorickettsia findlayensis* sp. nov.” The new taxon must be described as a new species, not as a *Candidatus*.

The description of “Candidatus *Neorickettsia finleia*” sp. nov. should be as follows.

**Description of *Neorickettsia findlayensis* sp. nov.** (find.lay.en**′**sis. N.L. fem. adj. *findlayensis*, from Findlay, Ohio, USA).

The species is pathogenic to horses. To date, all infected horses have originated from Findlay, OH, USA, and Ontario, Canada. Horse infection with *N. findlayensis* causes an illness characterized by fever, anorexia, depression, and diarrhea. *N. findlayensis* and *N. risticii* can be distinguished by PCR of *ssa3N*. *N. findlayensis* and *N. risticii* are serologically cross-reactive. *N. findlayensis* grows well in the P388D_1_ murine macrophage cell line. The type strain, Fin17, isolated from a horse from Ontario, Canada, in 2017, is available through the Centers for Disease Control and Prevention Rickettsial Isolate Reference Collection (CRIRC number NFI001^T^) and through the collection de Souches de l’Unite des Rickettsies (CSUR deposit ID Q1925).

