# Supplementary material for: Isolation and Molecular Analysis of a Novel Neorickettsia Species That Causes Potomac Horse Fever
Source: mBio. 2020 Feb 25;11(1):e03429-19. doi: 10.1128/mBio.03429-19 (PMC7042704; doi:10.1128/mBio.03429-19)
Supplement: FIG S1 [file mBio.03429-19-sf001.pdf]

Supplementary figure 1A - P51 external loop 2 aa sequence alignment

```
Majority                            VDANGKDIESSYNGXAS-D-SSNSVNNVKALSDGGILGDYTRNTDLFNKHKLSIEARRTLGNFAYGGFLEAEFSRKDAIA
                                    ---------+---------+---------+---------+---------+---------+---------+---------+
                                             10        20        30        40        50        60        70        80
                                    ---------+---------+---------+---------+---------+---------+---------+---------+
081                                 ..T......A....T.N---.T.--.-...........N.....................S.............K....S   74
Fin17                               ..T......A....T.N---.T.--.-...........N.....................S.............K....S   74
Tom16                               ..T......A....T.N---.T.--.-...........N.....................S.............K....S   74
Neorickettsia sp. from F. hepatica  ..S..NNV.A..S.NGT---D.S...G..P........N.....A.............A...............T.....   77
SF Oregon                           ..T......PT.A.TG.---D..-..S..P........N...S...............................K.G..S   76
SF Hirose                           ..T......PT.A.TS.---D..-..S..P........N...S...............................K.G..S   76
Dai17                               ..............TT.-EAN...........................................................   79
Dun17                               ..............TT.-EAN...........................................................   79
Tw2-1                               ..............TT.-EAN...........................................................   79
Too16                               ..............TT.-EAN...........................................................   79
Ont15                               -------.......TT.-EAN...........................................................   72
MN                                  ..............A.GN.A.........T..................................................   80
Reg16                               ..............A.GN.A.........T..................................................   80
Gab17                               ..............A.GN.A.....K...T..................................................   80
Luc17                               ..............A.GN.D.....K...T..................................................   80
Cup17                               ..............A.GN.A.....K...T..................................................   80
May17                               ..............A.GN.D.....K...T..................................................   80
Lad17                               ..............A..K.-............................................................   79
Herodia                             ..............A..K.-............................................................   79
Jan17                               ..............A..K.-............................................................   79
Illinois                            ..............A..K.-............................................................   79
Kawano                              ...S..........DS.---T.GT..G........V......S.....E............S.....L..........VS   77
Nakazaki                            ...S..........DS.---T.GT..G........V......S.....E............S.....L..........VS   77
Miyayama                            ...S..........DS.---T.GT..G........V......S.....E............S.....L..........VS   77

Majority                            ADNAYV
                                    ------

                                    ------
081                                 ......   80
Fin17                               ......   80
Tom16                               ......   80
Neorickettsia sp. from F. hepatica  ......   83
SF Oregon                           ......   82
SF Hirose                           ......   82
Dai17                               ......   85
Dun17                               ......   85
Tw2-1                               ......   85
Too16                               ......   85
Ont15                               ......   78
MN                                  ......   86
Reg16                               ......   86
Gab17                               ......   86
Luc17                               ......   86
Cup17                               ......   86
May17                               ......   86
Lad17                               ......   85
Herodia                             ......   85
Jan17                               ......   85
Illinois                            ......   85
Kawano                              ......   83
Nakazaki                            ......   83
Miyayama                            ......   83
```

Supplementary figure S1B - P51 external loop 2 aa Sequence Divergence and Identity

	Neorickettsia sp. 081	*Neorickettsia* sp. Fin 17	*Neorickettsia* sp. Tom 16	*Neorickettsia* sp. from *F. hepatica*	*Neorickettsia* sp. SF Oregon	*Neorickettsia* sp. SF Hirose	*N. risticii* Dai 17	*N. risticii* Dun 17	*N. risticii* Tw2-1	*N. risticii* Too 16	*N. risticii* MN	*N. risticii* Reg 16	*N. risticii* Gab 17	*N. risticii* Luc 17	*N. risticii* Cup 17	*N. risticii* May17	*N. risticii* Lad 17	*N. risticii* Herodia	*N. risticii* Jan 17	*N. risticii* Illinois	*N. sennetsu* Kawano	*N. sennetsu* Nakazaki	*N. sennetsu* Miyayama
Neorickettsia sp. 081		100.0	100.0	77.9	83.7	83.7	84.9	84.9	84.9	84.9	81.4	81.4	80.2	80.2	80.2	80.2	83.7	83.7	83.7	83.7	75.6	75.6	75.6
Neorickettsia sp. Fin 17	0.0		100.0	77.9	83.7	83.7	84.9	84.9	84.9	84.9	81.4	81.4	80.2	80.2	80.2	80.2	83.7	83.7	83.7	83.7	75.6	75.6	75.6
Neorickettsia sp. Tom 16	0.0	0.0		77.9	83.7	83.7	84.9	84.9	84.9	84.9	81.4	81.4	80.2	80.2	80.2	80.2	83.7	83.7	83.7	83.7	75.6	75.6	75.6
Neorickettsia sp. from *F. hepatica*	23.3	23.3	23.3		79.1	77.9	77.9	77.9	77.9	77.9	76.7	76.7	75.6	75.6	75.6	75.6	77.9	77.9	77.9	77.9	70.9	70.9	70.9
Neorickettsia SF Oregon	13.9	13.9	13.9	24.3		98.8	81.4	81.4	81.4	81.4	77.9	77.9	76.7	76.7	76.7	76.7	80.2	80.2	80.2	80.2	76.7	76.7	76.7
Neorickettsia SF Hirose	13.9	13.9	13.9	26.0	1.2		81.4	81.4	81.4	81.4	77.9	77.9	76.7	76.7	76.7	76.7	80.2	80.2	80.2	80.2	77.9	77.9	77.9
N. risticii Dai 17	10.8	10.8	10.8	24.0	17.9	17.9		100.0	100.0	100.0	91.9	91.9	90.7	89.5	90.7	89.5	93.0	93.0	93.0	93.0	81.4	81.4	81.4
N. risticii Dun 17	10.8	10.8	10.8	24.0	17.9	17.9	0.0		100.0	100.0	91.9	91.9	90.7	89.5	90.7	89.5	93.0	93.0	93.0	93.0	81.4	81.4	81.4
N. risticii Tw2-1	10.8	10.8	10.8	24.0	17.9	17.9	0.0	0.0		100.0	91.9	91.9	90.7	89.5	90.7	89.5	93.0	93.0	93.0	93.0	81.4	81.4	81.4
N. risticii Too 16	10.8	10.8	10.8	24.0	17.9	17.9	0.0	0.0	0.0		91.9	91.9	90.7	89.5	90.7	89.5	93.0	93.0	93.0	93.0	81.4	81.4	81.4
N. risticii MN	13.7	13.7	13.7	24.0	21.0	21.0	7.4	7.4	7.4	7.4		100.0	98.8	97.7	98.8	97.7	95.3	95.3	95.3	95.3	77.9	77.9	77.9
N. risticii Reg 16	13.7	13.7	13.7	24.0	21.0	21.0	7.4	7.4	7.4	7.4	0.0		98.8	97.7	98.8	97.7	95.3	95.3	95.3	95.3	77.9	77.9	77.9
N. risticii Gab 17	15.2	15.2	15.2	25.7	22.7	22.7	8.7	8.7	8.7	8.7	1.2	1.2		98.8	100.0	98.8	94.2	94.2	94.2	94.2	76.7	76.7	76.7
N. risticii Luc 17	15.2	15.2	15.2	25.7	22.7	22.7	10.1	10.1	10.1	10.1	2.4	2.4	1.2		98.8	100.0	94.2	94.2	94.2	94.2	76.7	76.7	76.7
N. risticii Cup 17	15.2	15.2	15.2	25.7	22.7	22.7	8.7	8.7	8.7	8.7	1.2	1.2	0.0	1.2		98.8	94.2	94.2	94.2	94.2	76.7	76.7	76.7
N. risticii May 17	15.2	15.2	15.2	25.7	22.7	22.7	10.1	10.1	10.1	10.1	2.4	2.4	1.2	0.0	1.2		94.2	94.2	94.2	94.2	76.7	76.7	76.7
N. risticii Lad 17	12.2	12.2	12.2	24.0	19.4	19.4	4.9	4.9	4.9	4.9	3.6	3.6	4.9	4.9	4.9	4.9		100.0	100.0	100.0	81.4	81.4	81.4
N. risticii Herodia	12.2	12.2	12.2	24.0	19.4	19.4	4.9	4.9	4.9	4.9	3.6	3.6	4.9	4.9	4.9	4.9	0.0		100.0	100.0	81.4	81.4	81.4
N. risticii Jan 17	12.2	12.2	12.2	24.0	19.4	19.4	4.9	4.9	4.9	4.9	3.6	3.6	4.9	4.9	4.9	4.9	0.0	0.0		100.0	81.4	81.4	81.4
N. risticii Illinois	12.2	12.2	12.2	24.0	19.4	19.4	4.9	4.9	4.9	4.9	3.6	3.6	4.9	4.9	4.9	4.9	0.0	0.0	0.0		81.4	81.4	81.4
N. sennetu Kawano	26.8	26.8	26.8	38.5	27.8	26.0	19.2	19.2	19.2	19.2	22.3	22.3	24.0	24.0	24.0	24.0	19.2	19.2	19.2	19.2		100.0	100.0
N. sennetsu Nakazaki	26.8	26.8	26.8	38.5	27.8	26.0	19.2	19.2	19.2	19.2	22.3	22.3	24.0	24.0	24.0	24.0	19.2	19.2	19.2	19.2	0.0		100.0
N. sennetsu Miyayama	26.8	26.8	26.8	38.5	27.8	26.0	19.2	19.2	19.2	19.2	22.3	22.3	24.0	24.0	24.0	24.0	19.2	19.2	19.2	19.2	0.0	0.0	

Percent Identity

Divergence
