# Supplementary material for: Isolation and Molecular Analysis of a Novel Neorickettsia Species That Causes Potomac Horse Fever
Source: mBio. 2020 Feb 25;11(1):e03429-19. doi: 10.1128/mBio.03429-19 (PMC7042704; doi:10.1128/mBio.03429-19)
Supplement: TABLE S1 [file mBio.03429-19-st001.pdf]

Table S1. Primers utilized for PCR amplification.

Primer[a]	Direction	Sequence (5′-3′)	Primer pair(s)	Gene(s) amplified	References
51K-F7	Forward	GTCTTCCAAAGATCGATGTCC	51K-R5	*p51 p2 external loop*	Gibson K et al., 2011 (41)
51K-R5	Reverse	TTCCGTAACCGGTTTCAAAG	51K-F7	*p51 p2 external loop*	Gibson K et al., 2011 (41)
PER51-7	Forward	TGTATAAACTTAGCAAGATATTAC	PER51-14, TM6	*p51 full sequence*	Gibson K et al., 2011 (41)
TM6	Reverse	CAGCGATGGAAGATACATC	PER51-7	*p51 full sequence*	Gibson K et al., 2011 (41)
PER51-14	Reverse	ACACTTGGTGTTAATGTAAGG	PER51-7	*p51 full sequence*	Gibson K et al., 2011 (41)
KM324	Forward	CCGGCTGTTGAAAAAACGACATCA	KM325	*p51 full sequence*	Gibson K et al., 2011 (41)
P51OT-F	Forward	TCTTTGAAACCGGTTACGGAAG	KM325	*p51 full sequence*	This study
KM325	Reverse	AGCTCATACGTGCTTCCAGTGATG	KM324, P51OT-F	*p51 full sequence*	Gibson K et al., 2011 (41)
838-1a	Forward	GGTAAGGATGAAGCAAAAGCAGTAC	838-4, Ssa1OT-R	*ssa1*	Gibson K et al., 2011 (41)
838-4	Reverse	CTGGTGCATAGTGCACTTCC	838-1a	*ssa1*	Gibson K et al., 2011 (41)
Ssa1OT-R	Reverse	TCCAAAGGCTTGTAGTTTTGTT	838-1a	*ssa1*	This study
ER-5-3	Forward	ATTTGAGAGTTTGATCCTGG	ER-3-2	*16S rRNA full sequence*	Chaichanasiriwithaya et al, 1994 (19)
ER-3-2	Reverse	GTTTTAAATGCAGTTCTTGG	ER-5-3	*16S rRNA full sequence*	Chaichanasiriwithaya et al, 1994 (19)
Eris-1	Forward	GGAATCAGGGCTGCTTGCAGCCT	Eris-2	*16S rRNA full sequence*	Kanter et al, 2000 (37)
Eris-2	Reverse	TGTGGGTACCGTCATTATCTTCCCCA	Eris-1	*16S rRNA full sequence*	Kanter et al, 2000 (37)
ER3	Forward	ATTTGAGAGTTTGATCCTGG	PC5, ER2	*16S rRNA full sequence*	Gibson K et al., 2011 (41)
PC5	Reverse	TACCTTGTTACGACTT	ER3	*16S rRNA full sequence*	Gibson K et al., 2011 (41)
ER2	Reverse	GTTTTAAATGCAGTTCTTGG	ER3	*16S rRNA full sequence*	Gibson K et al., 2011 (41)
ER2a	Forward	CCCGTAAGTTAGGTGTG	ER X	*16S rRNA full sequence*	Gibson K et al., 2011 (41)
ER X	Reverse	CATCTCACGACACGAGC	ER2a	*16S rRNA full sequence*	Gibson K et al., 2011 (41)
ER Y	Forward	CCAACACAGGTGTTGC	ER Z2	*16S rRNA full sequence*	Gibson K et al., 2011 (41)
ER Z2	Reverse	ACCCCAGTCACCCACCCC	ER Y	*16S rRNA full sequence*	Gibson K et al., 2011 (41)
840-1	Forward	CTAGTGCATCAAAAGGCGTGAG	840-2	*ssa3*	Gibson K et al., 2011 (41)
840-2	Reverse	CATTACCTGGACTTTCGAACAGC	840-1	*ssa3*	Gibson K et al., 2011 (41)
NCR839/840-1	Forward	CATAACTTAGGGCTACTATCCC	NCR840/841-1, 840-3	*ssa3*	Gibson K et al., 2011 (41)
840-3	Reverse	GTGAGAACATTGCCTACTTTATC	NCR839/840-1	*ssa3*	Gibson K et al., 2011 (41)
840-3F	Forward	GATAAAGTAGGCAATGTTCTCAC	NCR840/841-1	*ssa3*	Gibson K et al., 2011 (41)
NCR840/841-1	Reverse	CTTGTTATGGTAACCTGCTTG	NCR839/840-1, 840-3F	*ssa3*	Gibson K et al., 2011 (41)
