# Supplementary material for: Isolation and Molecular Analysis of a Novel Neorickettsia Species That Causes Potomac Horse Fever
Source: mBio. 2020 Feb 25;11(1):e03429-19. doi: 10.1128/mBio.03429-19 (PMC7042704; doi:10.1128/mBio.03429-19)
Supplement: FIG S2 [file mBio.03429-19-sf002.pdf]

Supplementary figue. 2A - Long P51 aa sequence alignment

```
Majority                           MYKLSKILLLTAALAGVAGASEVPLTEDQVPAVEKTTSNKPCVCNKTGPNQVKARLSKFADRCATGGXGSPGCSCNGSSD
                                   ---------+---------+---------+---------+---------+---------+---------+---------+
                                           10        20        30        40        50        60        70        80
                                   ---------+---------+---------+---------+---------+---------+---------+---------+
Neorickettsia sp. 081              --------------------------------------------------------------------------------     0
Neorickettsia sp. Fin17            ---------------...........G.S.......P...R.GLQE...............H.PA..M.....N.....S    65
Neorickettsia sp. Tom16            ---------------...........G.T.......P...R.GLQE...............H.PA..M.....N.....S    65
Neorickettsia sp. from F. hepatica ........F..L..TN......A...G.T..........RM......................SA..T.....H.....N    80
Neorickettsia SF oregon            .............FV...........G.P...........I.......................A..AS.L........S    80
Neorickettsia SF Hirose            .............FV...........G.P...........I.......................A..AS.L........S    80
N.risticii 90-12                   ...............................................................T...T............    80
N. risticii Illinois               ...............................................................T...T............    80
N. risticii 25-D                   ...............................................................T...T............    80
N. risticii PA-1                   -..............................................................T...T............    79
N. sennetsu Miyayama               ...........T...S..............................A..............H....-M..R....D....    79
N. sennetsu Kawano                 -..........T...S..............................A..............H....-M..R....D....    78
N. sennetsu Nakazaki               ...........T...S..............................A..............H....-M..R....D....    79

Majority                           PNGTDNCDAVNFVFKVKGSNDFSFGYGSNKDFFKLAKGLPKIDVLVDANGKDIESSYNGTXS---SSNXVNNVKALSDGG
                                   ---------+---------+---------+---------+---------+---------+---------+---------+
                                           90       100       110       120       130       140       150       160
                                   ---------+---------+---------+---------+---------+---------+---------+---------+
Neorickettsia sp. 081              ---------..................R.....E.............T......A.....AN---.T.---.........    65
Neorickettsia sp. Fin17            .S..E......................R.....E.............T......A.....AN---.T.---.........   139
Neorickettsia sp. Tom16            .S..E......................R.....E.............T......A.....AN---.T.---.........   139
Neorickettsia sp. from F. hepatica QSE........................R..G..E........N....S..NNV.A..S.NGT---D.SS..G..P.....   157
Neorickettsia SF oregon            TSE.....................A..K.QE..E.............T......PT.A..G.---D..-..S..P.....   156
Neorickettsia SF Hirose            TSE.....T...............A..K.QE..E.............T......PT.A..S.---D..-..S..P.....   156
N.risticii 90-12                   ...I..........R.............................................T.-EAN..S...........   159
N. risticii Illinois               ...I..........R.............................................T.NDA...S.K...T.....   160
N. risticii 25-D                   ...I..........R............................................AA.-KD...S...........   159
N. risticii PA-1                   ...I..........R.............................................T.-EAN..S...........   158
N. sennetsu Miyayama               L...S.....................A..Q..................S..........DS.---T.GT..G........   156
N. sennetsu Kawano                 L...S.....................A..Q..................S..........DS.---T.GT..G........   155
N. sennetsu Nakazaki               L...S.....................A..Q..................S..........DS.---T.GT..G........   156

Majority                           ILGDYTRNTDLFNKHKLSIEARRTLGNFAYGGFLEAEFSRKDAISADNAYVFFETGYGRFEMGRVSDSAVKPLRIDASSI
                                   ---------+---------+---------+---------+---------+---------+---------+---------+
                                          170       180       190       200       210       220       230       240
                                   ---------+---------+---------+---------+---------+---------+---------+---------+
Neorickettsia sp. 081              ...N.....................S.............K..........................E............V   145
Neorickettsia sp. Fin17            ...N.....................S.............K..........................E............V   219
Neorickettsia sp. Tom16            ...N.....................S.............K..........................E............V   219
Neorickettsia sp. from F. hepatica ...N.....A.............A...............T....A......................N....IK.....V   237
Neorickettsia SF oregon            ...N...S...............................K.G........................EN.....K.....V   236
Neorickettsia SF Hirose            ...N...S...............................K.G........................EN.....K.....V   236
N.risticii 90-12                   ............................................A.........................Q.I...V...   239
N. risticii Illinois               ............................................A.........................Q.I...V...   240
N. risticii 25-D                   ............................................A.........................Q.I...V...   239
N. risticii PA-1                   ............................................A.........................Q.I...V...   238
N. sennetsu Miyayama               V......S.....E............S.....L..........V....................IT....E.........   236
N. sennetsu Kawano                 V......S.....E............S.....L..........V....................IT....E.........   235
N. sennetsu Nakazaki               V......S.....E............S.....L..........V....................IT....E.........   236
```

```
Majority                           AAVGGGFNDLNWTTLANLEGRPIGATHSTTG-GSSTK-XSKRHKDAQHPFLVHANYYTQYNNPLRANFITTGLGNLRMAL
                                   ---------+---------+---------+---------+---------+---------+---------+---------+
                                           250       260       270       280       290       300       310       320
                                   ---------+---------+---------+---------+---------+---------+---------+---------+
Neorickettsia sp. 081              .......E...............N..RT.N---G..T-TCG............T..........................      221
Neorickettsia sp. Fin17            .......E...............N..RT.N---G..T-TCG............T..........................      295
Neorickettsia sp. Tom16            .......E...............N..RT.N---G..T-TCG............T..........................      295
Neorickettsia sp. from F. hepatica .......D...............D...TNGSNDT...-T.T............T..........................      316
Neorickettsia SF oregon            ......LD..............LH.E.AA----TG.P-KN...R.........TD.....E...................      311
Neorickettsia SF Hirose            ......LD..............LN.E.VA----TR.P-KN...R.........TD.....E...............Q...      311
N.risticii 90-12                   ..........S...........L.....-..T...S--Q......V.P.S....D..SP................F....      316
N. risticii Illinois               ..........S...........L.....-..T...S--Q......V.R......D...P.....................      317
N. risticii 25-D                   ..........S...........L...Y.-A.T...SS-Q......V.P.S....D..SP................F....      317
N. risticii PA-1                   ..........S...........L.....-..T...S--Q......V.R......D...P.....................      315
N. sennetsu Miyayama               .......G.......................N.D.Q.SS.T..R...R..........A.....................      316
N. sennetsu Kawano                 .......G.......................N.D.Q.SS.T..R...R..........A.....................      315
N. sennetsu Nakazaki               .......G.......................N.D.Q.SS.T..R...R..........A.....................      316

Majority                           GYTNSTADGTYHDIIDVGAGYAGKKGNLKYGISFSGQAGLSTPTGDEHHPLRRFEVGASAQIHTIKLAGSWGSTYLSGVK
                                   ---------+---------+---------+---------+---------+---------+---------+---------+
                                           330       340       350       360       370       380       390       400
                                   ---------+---------+---------+---------+---------+---------+---------+---------+
Neorickettsia sp. 081              ..............V.L..A...Q........                                                      253
Neorickettsia sp. Fin17            ..............V.L..A...Q.................A....V............V.L................L.      375
Neorickettsia sp. Tom16            ..............V.L..A...Q.................A....V............V.L................L.      375
Neorickettsia sp. from F. hepatica ..............L.L..A...Q.....................GL...........V...Q................E      396
Neorickettsia SF oregon            ........S.......M..A.T.Q....................A.I...........V...................IE      391
Neorickettsia SF Hirose            ........S.......M..A.T.Q......................I...........V...................IE      391
N.risticii 90-12                   ..S...........................P..........P......................................      396
N. risticii Illinois               ................M....V........A..........A......................................      397
N. risticii 25-D                   ..S.............M....VR.......P..........P......................................      397
N. risticii PA-1                   ..............................A..........A......................................      395
N. sennetsu Miyayama               ..............................A............................V.L..................      396
N. sennetsu Kawano                 ..............................A............................V.L..................      395
N. sennetsu Nakazaki               ..............................A............................V.L..................      396

Majority                           KLKDMQLDLSKAFADSSQLKATDGDSAYMTFGAAYEEGPVMFSLGYMESYNTFVKSVGVNTLRVVSLGTHYRITGSTYEL
                                   ---------+---------+---------+---------+---------+---------+---------+---------+
                                           410       420       430       440       450       460       470       480
                                   ---------+---------+---------+---------+---------+---------+---------+---------+
Neorickettsia sp. 081                                                                                                    253
Neorickettsia sp. Fin17            .-..................E...KH..............A...T...................................      454
Neorickettsia sp. Tom16            .-..................Q...KH..............A...T...................................      454
Neorickettsia sp. from F. hepatica .K.......N...T....T.T.....S.............A...N...................................      476
Neorickettsia SF oregon            .R.N...........G..IQE...SN..............A...N...................................      471
Neorickettsia SF Hirose            .R.N...........G..IQE...SN..............A...N...................................      471
N.risticii 90-12                   ...............N.......................................N........................      476
N. risticii Illinois               ...............N.......................................N........................      477
N. risticii 25-D                   ...............N.......................................N........................      477
N. risticii PA-1                   ...............N.......................................N........................      475
N. sennetsu Miyayama               .S.......T..........K.....T......T..............................................      476
N. sennetsu Kawano                 .S.......T..........K.....T......T..............................................      475
N. sennetsu Nakazaki               .S.......T..........K.....T......T..............................................      476

Majority                           TPYINTKCFMAQEAGIKXEDNNKGFVLTSGVKVSY
                                   ---------+---------+---------+-----
                                           490       500       510
                                   ---------+---------+---------+-----
Neorickettsia sp. 081                                                                                                    253
Neorickettsia sp. Fin17            ........                                                                              462
Neorickettsia sp. Tom16            ........                                                                              462
Neorickettsia sp. from F. hepatica .................S.................                                                   511
Neorickettsia SF oregon            ..........L......S.E...............                                                   506
Neorickettsia SF Hirose            ..........L......S.E...............                                                   506
N.risticii 90-12                   .............T...A.................                                                   511
N. risticii Illinois               .............T...A.................                                                   512
N. risticii 25-D                   .............T...A.................                                                   512
N. risticii PA-1                   ........                                                                              483
N. sennetsu Miyayama               .................A.........A.......                                                   511
N. sennetsu Kawano                 .................                                                                     492
N. sennetsu Nakazaki               .................A.........A.......                                                   511
```

Supplementary figure S2B – Long P51 aa sequence Divergence and identity

	Neorickettsia sp. Fin 17	*Neorickettsia* sp. Tom 16	*Neorickettsia* sp. from *F. hepatica*	*Neorickettsia* SF Hirose	*Neorickettsia* SF Oregon	*N. risticii* 90-12	*N. risticii* Illinois	*N. risticii* 25-D	*N. risticii* PA-1	*N. sennetsu* Miyayama	*N sennetsu* Kawano	*N. sennetsu* Nakazaki	*N. helminthoeca*
Neorickettsia sp. Fin 17		99.6	85.3	85.0	85.0	84.1	84.8	83.7	85.2	83.3	83.3	83.3	46.5
Neorickettsia sp. Tom 16	0.4		85.5	84.8	84.8	84.1	84.8	83.7	85.2	83.3	83.3	83.3	46.5
Neorickettsia sp. from *F. hepatica*	16.4	16.2		84.5	84.9	82.0	82.4	81.2	82.9	80.3	80.3	80.3	46.5
Neorickettsia SF Hirose	16.7	17.0	17.4		98.5	81.8	82.7	81.2	82.7	80.1	80.1	80.1	46.8
Neorickettsia SF Oregon	16.7	17.0	16.9	1.5		82.0	82.9	81.4	82.9	80.1	80.1	80.1	46.6
N. risticii 91-12	17.9	17.9	20.6	20.9	20.6		97.2	97.9	98.5	87.1	87.1	87.1	45.6
N. risticii Illinois	17.1	17.1	20.1	19.7	19.5	2.8		96.6	98.7	87.6	87.6	87.6	46.0
N. risticii 25-D	18.4	18.4	21.7	21.7	21.4	2.2	3.5		96.4	85.9	85.9	85.9	45.3
N. risticii PA-1	16.5	16.5	19.5	19.7	19.5	1.5	1.3	3.7		88.4	88.4	88.4	45.8
N. sennetsu Miyayama	18.9	18.9	22.9	23.1	23.1	14.2	13.6	15.7	12.6		100.0	100.0	45.2
N. sennetsu Kawano	18.9	18.9	22.9	23.1	23.1	14.2	13.6	15.7	12.6	0.0		100.0	45.2
N. sennetsu Nakazaki	18.9	18.9	22.9	23.1	23.1	14.2	13.6	15.7	12.6	0.0	0.0		45.2
N. helminthoeca	89.7	89.7	89.7	88.7	89.3	92.4	91.1	93.4	91.7	93.8	93.8	93.8	

Percent Identity

Divergence
