# Supplementary material for: Isolation and Molecular Analysis of a Novel Neorickettsia Species That Causes Potomac Horse Fever
Source: mBio. 2020 Feb 25;11(1):e03429-19. doi: 10.1128/mBio.03429-19 (PMC7042704; doi:10.1128/mBio.03429-19)
Supplement: FIG S3 [file mBio.03429-19-sf003.pdf]

Supplementary figure. 3A- 16S rRNA gene base sequence alignment

```
Majority                            ---------------GATCXTGGCTCAGAACGAACGCTAGCGGTAGGCTTAACACATGCAAGTCGAACGGAATCAAGGCT
                                    ---------+---------+---------+---------+---------+---------+---------+---------+
                                            10        20        30        40        50        60        70        80
                                    ---------+---------+---------+---------+---------+---------+---------+---------+
Fin17                               -----------------------...............CC....................................A...     57
Tom16                               -----------------------...............CC....................................A...     57
081                                 CTGATTTGAGAGTTT....C........................................................A...     80
Neorickettsia from F. hepatica      CTGATTTGAGAGTTT....C........................................................A...     80
SF Oregon                           ----------------------------------------------------------......................     22
SF Hirose                           ---------------....A............................................................     65
N. risticii Illinois                CTGATTTGAGAGTTT....C.......................................................G....     80
16S rRNA Pennsylvaisa               -----------------------....................................................G....     57
16S rRNA isolated from horse 1      ---ATTTGAGAGTTT....C.......................................................G....     77
Neorickettsia from Lecithodendridae ----------------------------------------------------------......................     22
N. sennetsu Miyayama                CTGATTTGAGAGTTT....C........................................................A...     80

Majority                            GCTTGCAGCTTTGGTCCCGTGGCAGACGGGTGCGTAACGCGTGGGAACTTGCCTGGTAGTAGGGAATAACCAGTGGAAAC
                                    ---------+---------+---------+---------+---------+---------+---------+---------+
                                            90       100       110       120       130       140       150       160
                                    ---------+---------+---------+---------+---------+---------+---------+---------+
Fin17                               ...........C....................................................................    137
Tom16                               ...........C....................................................................    137
081                                 ...........C....................................................................    160
Neorickettsia from F. hepatica      ...........C....................................................................    160
SF Oregon                           ................................................................................    102
SF Hirose                           ................................................................................    145
N. risticii Illinois                .........C......................................................................    160
16S rRNA Pennsylvaisa               .........C......................................................................    137
16S rRNA isolated from horse 1      .........C......................................................................    157
Neorickettsia from Lecithodendridae .........C...A..A.................................A.............................    102
N. sennetsu Miyayama                .........C......................................................................    160

Majority                            ATTGGGCAACACCCTATACGCCCTGAGGGGGAAAATTTATTGCTATCAGATAGGCCCGCGTTAGATTAGCTAGTTGGTGG
                                    ---------+---------+---------+---------+---------+---------+---------+---------+
                                           170       180       190       200       210       220       230       240
                                    ---------+---------+---------+---------+---------+---------+---------+---------+
Fin17                               ................................................................................    217
Tom16                               ................................................................................    217
081                                 ................................................................................    240
Neorickettsia from F. hepatica      ................................................................................    240
SF Oregon                           .....A..........................................................................    182
SF Hirose                           .....A..........................................................................    225
N. risticii Illinois                ................................................................................    240
16S rRNA Pennsylvaisa               ................................................................................    217
16S rRNA isolated from horse 1      ................................................................................    237
Neorickettsia from Lecithodendridae ................................................................................    182
N. sennetsu Miyayama                ................................................................................    240
```

```
Majority                            GGTAATGGCCTACCAAGGCGACGATCTATAGCTGGTCTGAGAGGATGATCAGCCACACTGGAACTGAGACACGGTCCAGA
                                    ---------+---------+---------+---------+---------+---------+---------+---------+
                                           250       260       270       280       290       300       310       320
                                    ---------+---------+---------+---------+---------+---------+---------+---------+
Fin17                               ................................................................................     297
Tom16                               ................................................................................     297
081                                 ................................................................................     320
Neorickettsia from F. hepatica      ................................................................................     320
SF Oregon                           .....C..........................................................................     262
SF Hirose                           .....C..........................................................................     305
N. risticii Illinois                ................................................................................     320
16S rRNA Pennsylvaisa               ................................................................................     297
16S rRNA isolated from horse 1      ................................................................................     317
Neorickettsia from Lecithodendridae ................................................................................     262
N. sennetsu Miyayama                ................................................................................     320

Majority                            CTCCTACGGGAGGCAGCAGTGAGGAATATTGGACAATGGGCGAAAGCCTGATCCAGCCATACCGCATGAGTGATGAAGGC
                                    ---------+---------+---------+---------+---------+---------+---------+---------+
                                           330       340       350       360       370       380       390       400
                                    ---------+---------+---------+---------+---------+---------+---------+---------+
Fin17                               ................................................................................     377
Tom16                               ................................................................................     377
081                                 ................................................................................     400
Neorickettsia from F. hepatica      ................................................................................     400
SF Oregon                           ................................................................................     342
SF Hirose                           ................................................................................     385
N. risticii Illinois                ................................................................................     400
16S rRNA Pennsylvaisa               ................................................................................     377
16S rRNA isolated from horse 1      ................................................................................     397
Neorickettsia from Lecithodendridae ................................................................................     342
N. sennetsu Miyayama                ................................................................................     400

Majority                            CCTTGGGTTGTAAAGCTCTTTTAGTGGGGAAGATAATGACGGTACCCACAGAAGAAGTCCCGGCTAACTCCGTGCCAGCA
                                    ---------+---------+---------+---------+---------+---------+---------+---------+
                                           410       420       430       440       450       460       470       480
                                    ---------+---------+---------+---------+---------+---------+---------+---------+
Fin17                               ................................................................................     457
Tom16                               ................................................................................     457
081                                 ................................................................................     480
Neorickettsia from F. hepatica      ................................................................................     480
SF Oregon                           ................................................................................     422
SF Hirose                           ................................................................................     465
N. risticii Illinois                ................................................................................     480
16S rRNA Pennsylvaisa               ................................................................................     457
16S rRNA isolated from horse 1      ................................................................................     477
Neorickettsia from Lecithodendridae ................................................................................     422
N. sennetsu Miyayama                ...................................G............................................     480

Majority                            GCCGCGGTAATACGGAGGGGGCTAGCGTTGTTCGGAATTACTGGGCGTAAAGGGTGCGTAGGCGGTCCCGTAAGTTAGGT
                                    ---------+---------+---------+---------+---------+---------+---------+---------+
                                           490       500       510       520       530       540       550       560
                                    ---------+---------+---------+---------+---------+---------+---------+---------+
Fin17                               ................................................................................     537
Tom16                               ................................................................................     537
081                                 ................................................................................     560
Neorickettsia from F. hepatica      ................................................................................     560
SF Oregon                           ................................................................................     502
SF Hirose                           ................................................................................     545
N. risticii Illinois                ................................................................................     560
16S rRNA Pennsylvaisa               ................................................................................     537
16S rRNA isolated from horse 1      ................................................................................     557
Neorickettsia from Lecithodendridae ................................................................................     502
N. sennetsu Miyayama                ................................................................................     560
```

```
Majority                            GTGAAATCCTTGGGCTTAACCCAAGAACTGCATTTAAAACTGTGGGACTCGAACGTGATAGAGGGCAATGGAATTTTTGG
                                    ---------+---------+---------+---------+---------+---------+---------+---------+
                                           570       580       590       600       610       620       630       640
                                    ---------+---------+---------+---------+---------+---------+---------+---------+
Fin17                               ......................T.........................................................  617
Tom16                               ......................T.........................................................  617
081                                 ................................................................................  640
Neorickettsia from F. hepatica      ................................................................................  640
SF Oregon                           ................................................................................  582
SF Hirose                           ................................................................................  625
N. risticii Illinois                ..........................................................G.....................  640
16S rRNA Pennsylvaisa               ..........................................................G.....................  617
16S rRNA isolated from horse 1      ..........................................................G.....................  637
Neorickettsia from Lecithodendridae ..........................................................G.....................  582
N. sennetsu Miyayama                .......................G..................C...............G.....................  640

Majority                            TGTAGGGGTGAAATCCGTAGATATCAAGAGGAACGTCAGGGGCGAAAGCGATTGCCTGGATCACAGTTGACGCTGAGGCA
                                    ---------+---------+---------+---------+---------+---------+---------+---------+
                                           650       660       670       680       690       700       710       720
                                    ---------+---------+---------+---------+---------+---------+---------+---------+
Fin17                               ................................................................................  697
Tom16                               ................................................................................  697
081                                 ................................................................................  720
Neorickettsia from F. hepatica      ................................................................................  720
SF Oregon                           ................................................................................  662
SF Hirose                           ................................................................................  705
N. risticii Illinois                ................................................................................  720
16S rRNA Pennsylvaisa               ................................................................................  697
16S rRNA isolated from horse 1      ................................................................................  717
Neorickettsia from Lecithodendridae ................................................................................  662
N. sennetsu Miyayama                ................................................................................  720

Majority                            CGAAAGCGTGGGGAGCAAACAGGATTAGATACCCTGGTAGTCCACGCTGTAAACGATGAGTGTTAAAAGTGGGTTATTTT
                                    ---------+---------+---------+---------+---------+---------+---------+---------+
                                           730       740       750       760       770       780       790       800
                                    ---------+---------+---------+---------+---------+---------+---------+---------+
Fin17                               ................................................................................  777
Tom16                               ................................................................................  777
081                                 ................................................................................  800
Neorickettsia from F. hepatica      ................................................................................  800
SF Oregon                           ................................................................................  742
SF Hirose                           ................................................................................  785
N. risticii Illinois                ................................................................................  800
16S rRNA Pennsylvaisa               ................................................................................  777
16S rRNA isolated from horse 1      ................................................................................  797
Neorickettsia from Lecithodendridae ................................................................................  742
N. sennetsu Miyayama                ................................................................................  800

Majority                            ATCTGCTTTGTAGCTAACGCGTTAAACACTCCGCCTGGGGACTACGGTCGCAAGACTAAAACTCAAAGGAATTGACGGGG
                                    ---------+---------+---------+---------+---------+---------+---------+---------+
                                           810       820       830       840       850       860       870       880
                                    ---------+---------+---------+---------+---------+---------+---------+---------+
Fin17                               ................................................................................  857
Tom16                               ................................................................................  857
081                                 ................................................................................  880
Neorickettsia from F. hepatica      ................................................................................  880
SF Oregon                           ................................................................................  822
SF Hirose                           ................................................................................  865
N. risticii Illinois                ................................................................................  880
16S rRNA Pennsylvaisa               ................................................................................  857
16S rRNA isolated from horse 1      ................................................................................  877
Neorickettsia from Lecithodendridae ................................................................................  822
N. sennetsu Miyayama                ................................................................................  880
```

```
Majority                            ACTCGCACAAGCGGTGGAGTATGTGGTTTAATTCGATGCAACGCGAAAAACCTTACCATACCTTGACATGTGGATCGTAT
                                    ---------+---------+---------+---------+---------+---------+---------+---------+
                                             890       900       910       920       930       940       950       960
                                    ---------+---------+---------+---------+---------+---------+---------+---------+
Fin17                               ................................................................................      937
Tom16                               ................................................................................      937
081                                 ................................................................................      960
Neorickettsia from F. hepatica      ................................................................................      960
SF Oregon                           ................................................................................      902
SF Hirose                           ................................................................................      945
N. risticii Illinois                ...........................................................................T....      960
16S rRNA Pennsylvaisa               ...........................................................................T....      937
16S rRNA isolated from horse 1      ...........................................................................T....      957
Neorickettsia from Lecithodendridae ................................................................................      902
N. sennetsu Miyayama                ................................................................................      960

Majority                            CCCTCTGAAGGGAGGGAGTCAGTTCGGCTGGATCCAACACAGGTGTTGCATGGCTGTCGTCAGCTCGTGTCGTGAGATGT
                                    ---------+---------+---------+---------+---------+---------+---------+---------+
                                             970       980       990       1000      1010      1020      1030      1040
                                    ---------+---------+---------+---------+---------+---------+---------+---------+
Fin17                               ..........A.....................................................................     1017
Tom16                               ..........A.....................................................................     1017
081                                 ..........A.....................................................................     1040
Neorickettsia from F. hepatica      ................................................................................     1040
SF Oregon                           ........................A.......................................................      982
SF Hirose                           ........................A.......................................................     1025
N. risticii Illinois                ................................................................................     1040
16S rRNA Pennsylvaisa               ................................................................................     1017
16S rRNA isolated from horse 1      ................................................................................     1037
Neorickettsia from Lecithodendridae ................................................................................      982
N. sennetsu Miyayama                ................................................................................     1040

Majority                            TGGGTTAAGTCCCGCAACGAGCGCAACCCTCATCCTTAGTTGCCAGCGGTTCGGCCGGGAACTTTAAGGAAACTGCCAGT
                                    ---------+---------+---------+---------+---------+---------+---------+---------+
                                             1050      1060      1070      1080      1090      1100      1110      1120
                                    ---------+---------+---------+---------+---------+---------+---------+---------+
Fin17                               ................................................................................     1097
Tom16                               ................................................................................     1097
081                                 ................................................................................     1120
Neorickettsia from F. hepatica      ................................................................................     1120
SF Oregon                           ................................................................................     1062
SF Hirose                           ................................................................................     1105
N. risticii Illinois                ................................................................................     1120
16S rRNA Pennsylvaisa               ................................................................................     1097
16S rRNA isolated from horse 1      ...-............................................................................     1116
Neorickettsia from Lecithodendridae ................................................................................     1062
N. sennetsu Miyayama                ................................................................................     1120

Majority                            GACAAGCTGGAGGAAGGTGGGGACGACGTCAAGTCATCATGGCCCTTATGGTATGGGCTACACACGTACTACAATGGCAA
                                    ---------+---------+---------+---------+---------+---------+---------+---------+
                                             1130      1140      1150      1160      1170      1180      1190      1200
                                    ---------+---------+---------+---------+---------+---------+---------+---------+
Fin17                               ................................................................................     1177
Tom16                               ................................................................................     1177
081                                 ................................................................................     1200
Neorickettsia from F. hepatica      ................................................................................     1200
SF Oregon                           ................................................................................     1142
SF Hirose                           ................................................................................     1185
N. risticii Illinois                ................................................................................     1200
16S rRNA Pennsylvaisa               ................................................................................     1177
16S rRNA isolated from horse 1      ................................................................................     1196
Neorickettsia from Lecithodendridae ................................................................................     1142
N. sennetsu Miyayama                ................................................................................     1200
```

```
Majority                           CTACAATGAGCTAGCTACACCGTAAGGTGATGCCAATCTCTTAAAAGTTGTCTCAGTACGGATTGCCTTCTGCAACTCGA
                                   ---------+---------+---------+---------+---------+---------+---------+---------+
                                         1210      1220      1230      1240      1250      1260      1270      1280
                                   ---------+---------+---------+---------+---------+---------+---------+---------+
Fin17                              ....................T...........................................................   1257
Tom16                              ....................T...........................................................   1257
081                                ....................T...........................................................   1280
Neorickettsia from F. hepatica     ................................................................................   1280
SF Oregon                          ......................C..................................T......................   1222
SF Hirose                          ......................C..................................T......................   1265
N. risticii Illinois               ..............................C..............G..................................   1280
16S rRNA Pennsylvaisa              ..............................C..............G..................................   1257
16S rRNA isolated from horse 1     ..............................C..............G..................................   1276
Neorickettsia from Lecithodendridae ..............................C.................................................   1222
N. sennetsu Miyayama               ........G........................A......C.......................................   1280

Majority                           AGGCATGAAGTTGGAATCGCTAGTAATCGCAGATCAGCATGCTGCGGTGAATACGTTCTCGGGTCTTGTACACACTGCCC
                                   ---------+---------+---------+---------+---------+---------+---------+---------+
                                         1290      1300      1310      1320      1330      1340      1350      1360
                                   ---------+---------+---------+---------+---------+---------+---------+---------+
Fin17                              ................................................................................   1337
Tom16                              ................................................................................   1337
081                                ................................................................................   1360
Neorickettsia from F. hepatica     ................................................................................   1360
SF Oregon                          ................................................................................   1302
SF Hirose                          ................................................................................   1345
N. risticii Illinois               ................................................................................   1360
16S rRNA Pennsylvaisa              ................................................................................   1337
16S rRNA isolated from horse 1     ................................................................................   1356
Neorickettsia from Lecithodendridae ................................................................................   1302
N. sennetsu Miyayama               ................................................................................   1360

Majority                           GTCAGGCCATGGGAGTTCATCTTACTCGAAGCTAGTGAGCTGACCGCAAGGAGGCAGCTATCTACGGTGGGGTGGGTGAC
                                   ---------+---------+---------+---------+---------+---------+---------+---------+
                                         1370      1380      1390      1400      1410      1420      1430      1440
                                   ---------+---------+---------+---------+---------+---------+---------+---------+
Fin17                              ....................................................................               1405
Tom16                              ....................................................................               1405
081                                ................................................................................   1440
Neorickettsia from F. hepatica     ................................................................................   1440
SF Oregon                          ..............................                                                     1332
SF Hirose                          .........................................A..........A...........................   1425
N. risticii Illinois               ................................................................................   1440
16S rRNA Pennsylvaisa              ................................................................................   1417
16S rRNA isolated from horse 1     ................................................................................   1436
Neorickettsia from Lecithodendridae ..............................                                                    1332
N. sennetsu Miyayama               ................................................................................   1440

Majority                           TGGGGTTAAGTCXXXXXXXXXXXXXXXXXXXXXXXXXXXXXXXXXXXXXXXXXXXXXX
                                   ---------+---------+---------+---------+---------+--------
                                         1450      1460      1470      1480      1490
                                   ---------+---------+---------+---------+---------+--------
Fin17                                                                                                                 1405
Tom16                                                                                                                 1405
081                                ............GTAACAAGGTAGCCGTAGGTGAACCTGCGGCTGGATTACCTCCTTT                         1498
Neorickettsia from F. hepatica     ............GTAACAAGGTAGCCGTAGGTGAACCTGCGGCTGGATTACCTCCTTT                         1498
SF Oregon                                                                                                             1332
SF Hirose                          ..........                                                                         1435
N. risticii Illinois               ............GTAACAAGGTAGCCGTAGGTGAACCTGCGGCTGGATTACCTCCTTT                         1498
16S rRNA Pennsylvaisa              ............GTAACAAGGTAGCCGTAGGTGAACCT                                             1455
16S rRNA isolated from horse 1     ............                                                                       1448
Neorickettsia from Lecithodendridae                                                                                   1332
N. sennetsu Miyayama               ............GTAACAAGGTAGCCGTAGGTGAACCTGCGGCTGGATTACCTCCTTT                         1498
```

Supplementary figure 3B – 16S rRNA gene base sequence divergence and identity

Percent Identity (upper right); Divergence (lower left)

	Neorickettsia sp. Fin 17	*Neorickettsia* sp. Tom 16	*Neorickettsia* sp. 081	*Neorickettsia* SF Hirose	*Neorickettsia* SF Oregon	*N. risticii* Illinois	*N. risticii* Pennsylvania	*N. risticii* Horse 1	*N. sennetsu* Miyayama	*N. helminthoeca* Oregon
Neorickettsia sp. Fin 17		100.0	99.9	99.2	99.2	99.2	99.2	99.2	99.1	95.4
Neorickettsia sp. Tom 16	0.0		99.9	99.2	99.2	99.2	99.2	99.2	99.1	95.4
Neorickettsia sp. 081	0.1	0.1		99.3	99.3	99.2	99.2	99.2	99.2	95.4
Neorickettsia SF Hirose	0.8	0.8	0.7		100.0	99.2	99.2	99.2	98.9	95.2
Neorickettsia SF Oregon	0.8	0.8	0.7	0.0		99.2	99.2	99.2	98.9	95.2
N. risticii Illinois	0.8	0.8	0.8	0.8	0.8		100.0	100.0	99.2	95.5
N. risticii Pennsylvania	0.8	0.8	0.8	0.8	0.8	0.0		100.0	99.2	95.5
N. risticii Horse 1	0.8	0.8	0.8	0.8	0.8	0.0	0.0		99.2	95.5
N. sennetsu Miyayama	0.9	0.9	0.8	1.1	1.1	0.8	0.8	0.8		95.4
N. helminthoeca Oregon	4.8	4.8	4.8	5.0	5.0	4.6	4.6	4.6	4.8	
