# Supplementary material for: Isolation and Molecular Analysis of a Novel Neorickettsia Species That Causes Potomac Horse Fever
Source: mBio. 2020 Feb 25;11(1):e03429-19. doi: 10.1128/mBio.03429-19 (PMC7042704; doi:10.1128/mBio.03429-19)
Supplement: FIG S4 [file mBio.03429-19-sf004.pdf]

Supplementary figure. 4 - Ssa-3 N-terminal two aa sequence repeats alignment

```
Majority              QAKAQNVVAVLKDSIAKAVLESDKFKALLQTQGKAELQDLLTNDNFKGLFEDQAKAQNVVAVLKDSIAKAVLESDKFKAL
                      ---------+---------+---------+---------+---------+---------+---------+---------+
                               10        20        30        40        50        60        70        80
                      ---------+---------+---------+---------+---------+---------+---------+---------+
N. risticii Illinois  ................................................................................   80
N. risticii Herodia   .................T..............................................................   80
Dai17                 ................................................................................   80
May17                 ................................................................................   80
Jun17                 ................................................................................   80
Cup17                 ................................................................................   80
Dun17                 .....................................................................T..........   80
Lad17                 ................................................................................   80
Too16                 .......................................V..SE....................................   80
N. risticii OV        .......A......V..T..G...........................................................   80
Luc17                 ................................................................................   80
Reg16                 ................................................................................   80
Gab17                 ................................................................................   80
N. risticii MN        ................................................................................   80
081                   .......A......A............................E...............A......A.............   80
Fin17                 .......A......A............................E...............A......A.............   80
Tom16                 .......A......A............................E...............A......A.............   80

Majority              LQTQGKAELQDLLTNDNFKGLFED
                      ---------+---------+----
                               90       100
                      ---------+---------+----
N. risticii Illinois  ........................   104
N. risticii Herodia   ........................   104
Dai17                 ........................   104
May17                 ........................   104
Jun17                 ........................   104
Cup17                 ........................   104
Dun17                 ........................   104
Lad17                 ........................   104
Too16                 ........................   104
N. risticii OV        ........................   104
Luc17                 ........................   104
Reg16                 ........................   104
Gab17                 ........................   104
N. risticii MN        ........................   104
081                   ...............E........   104
Fin17                 ...............E........   104
Tom16                 ...............E........   104
```
