# Supplementary material for: Isolation and Molecular Analysis of a Novel Neorickettsia Species That Causes Potomac Horse Fever
Source: mBio. 2020 Feb 25;11(1):e03429-19. doi: 10.1128/mBio.03429-19 (PMC7042704; doi:10.1128/mBio.03429-19)
Supplement: FIG S5 [file mBio.03429-19-sf005.pdf]

Supplementary figure. S5 - Ssa1 aa sequence alignment

```
Majority                           GKDEAKAVLTHEKFKDLFN------------DKT------------------------------------------TAG-
                                   ---------+---------+---------+---------+---------+---------+---------+---------+
                                           10        20        30        40        50        60        70        80
                                   ---------+---------+---------+---------+---------+---------+---------+---------+
N. risticii Tool6                  R..................------------...------------------------------------------...-      25
N. risticii Ont15                  ----------------------------------------------------------------------------...-       3
N. risticii PA-1                   ...................------------...------------------------------------------...-      25
N. risticii Cup17                  ..........D....G..D------------...------------------------------------------...-      25
N. risticii Gab17                  ..........D....G..D------------...------------------------------------------...-      25
N. risticii OH07-1                 ...................------------...------------------------------------------...-      25
N. risticii Reg16                  --RIT..............------------...------------------------------------------...-      23
N. risticii Lad17                  ...................------------...------------------------------------------...-      25
N. risticii Jan17                  ----...............------------...------------------------------------------...-      21
N. risticii Luc17                  ...................------------...------------------------------------------...-      25
N. risticii May17                  ...................------------...------------------------------------------...-      25
MI (US patent)                     ..TQV.E...N....E..GSDGKDILKDILT.N.GNFKGLIESTGKAQVKEVLTNEKFKELFGSEGKDILKEILTDNT.N      80
N. risticii Illinois               ..A.V.E...N....E..GSDGKDILKDILT.S.G---------------------KFKELIGSSGKDILKNILTDST.-      58
N. sennetsu Miyayama               ..VQ..D...N....E.IE------------SAG------------------------------------------KD--      24
Neorickettsia sp. from F. hepatica ..EKV.DL..D.N..K..E------------.D.------------------------------------------K.N-      25
OR (US patetnt)                    ..EKV..L..D.N..K..E------------.D.------------------------------------------K.N-      25

Majority                           ------------------------------------------------------YVKEILTSDKFKXLF---------ED
                                   ---------+---------+---------+---------+---------+---------+---------+---------+
                                           90       100       110       120       130       140       150       160
                                   ---------+---------+---------+---------+---------+---------+---------+---------+
N. risticii Tool6                  ------------------------------------------------------............E..---------T.      42
N. risticii Ont15                  ------------------------------------------------------............E..---------T.      20
N. risticii PA-1                   ------------------------------------------------------............E..---------T.      42
N. risticii Cup17                  ------------------------------------------------------............E..---------T.      42
N. risticii Gab17                  ------------------------------------------------------............E..---------T.      42
N. risticii OH07-1                 ------------------------------------------------------............E..---------T.      42
N. risticii Reg16                  ------------------------------------------------------............K..-----------      40
N. risticii Lad17                  ------------------------------------------------------............K..---------..      42
N. risticii Jan17                  ------------------------------------------------------............K..---------..      38
N. risticii Luc17                  ------------------------------------------------------............K..---------..      42
N. risticii May17                  ------------------------------------------------------............K..---------..      42
MI (US patent)                     FKGLIEGKGKDEAKGVLTDEKFKGLFDDKTIAGYVKEILTSEKFKKLFENGGKEK...L.IDEN..K..---------..     151
N. risticii Illinois               ------------------------------------------KFKELIESAGKGKL.DL.IDGN..K..---------..      87
N. sennetsu Miyayama               ------------------------------------------------------K...L..DEN..K..---------..      41
Neorickettsia sp. from F. hepatica ------------------------------------------------------N...V..DTNAAEIL---------K.      42
OR (US patetnt)                    ------------------------------------------------------H...V..DTNA.EILTDQTGKEVLKN      51
```

```
Majority                            XT-----KAGYVKEILTNDTAKEILTDQTAKEVLK---------------------------------------------
                                    ---------+---------+---------+---------+---------+---------+---------+---------+
                                            170       180       190       200       210       220       230       240
                                    ---------+---------+---------+---------+---------+---------+---------+---------+
N. risticii Too16                   A.-----............................---------------------------------------------     72
N. risticii Ont15                   A.-----............................---------------------------------------------     50
N. risticii PA-1                    A.-----............................---------------------------------------------     72
N. risticii Cup17                   A.-----..........SE.------------------------------------------------------------     57
N. risticii Gab17                   A.-----..........SE.------------------------------------------------------------     57
N. risticii OH07-1                  A.-----............................---------------------------------------------     72
N. risticii Reg16                   N.-----............................---------------------------------------------     70
N. risticii Lad17                   N.-----............................---------------------------------------------     72
N. risticii Jan17                   N.-----............................---------------------------------------------     68
N. risticii Luc17                   N.-----............................---------------------------------------------     72
N. risticii May17                   N.-----............................---------------------------------------------     72
MI (US patent)                      D.-----..AH......DSN......NEV......SDKFKDAITGAGKDALKEILTCDKFKDAVTGNGKDALKEILTCDK    226
N. risticii Illinois                D.-----..AH......DSN......NEV......SDKFKDAITGAGKDALKEILTCDKFKEAVTGDGKDALKEILTCDK    162
N. sennetsu Miyayama                N.-----...H...V..DTN......N........---------------------------------------------     71
Neorickettsia sp. from F. hepatica  S.AKEVL.DTNA.....DQ...DV.K.G.......---------------------------------------------     77
OR (US patetnt)                     S.AKDIL.STNAA.V.KDAN.........G.....---------------------------------------------     86

Majority                            ---------------------------------------DST------------------------------------AK
                                    ---------+---------+---------+---------+---------+---------+---------+---------+
                                            250       260       270       280       290       300       310       320
                                    ---------+---------+---------+---------+---------+---------+---------+---------+
N. risticii Too16                   ---------------------------------------.G.------------------------------------..     77
N. risticii Ont15                   ---------------------------------------.G.------------------------------------..     55
N. risticii PA-1                    ---------------------------------------.G.------------------------------------..     77
N. risticii Cup17                   --------------------------------------------------------------------------------     57
N. risticii Gab17                   --------------------------------------------------------------------------------     57
N. risticii OH07-1                  ---------------------------------------.G.------------------------------------..     77
N. risticii Reg16                   ---------------------------------------..---------------------------------------     72
N. risticii Lad17                   ---------------------------------------...------------------------------------..     77
N. risticii Jan17                   ---------------------------------------...------------------------------------..     73
N. risticii Luc17                   ---------------------------------------...------------------------------------..     77
N. risticii May17                   ---------------------------------------...------------------------------------..     77
MI (US patent)                      FKEAVTGDGKDKLKEILTHEKFKALIESEGKDILKDILT...GKFKELIESTGKDKLKEILVDEKFKALFTDATKAGYV.    306
N. risticii Illinois                FKDAVTGNGKDKLKEILTHEKFKALIESEGKDILKDILT...GKFKELIESTGKDEAKAVLTDEKFKDLFNDKTTAGYV.    242
N. sennetsu Miyayama                ---------------------------------------ND.------------------------------------..     76
Neorickettsia sp. from F. hepatica  ---------------------------------------NT.------------------------------------..     82
OR (US patetnt)                     ---------------------------------------N..------------------------------------..     91

Majority                            DILKDTNAAALLK-------------------------------D-----------STAKEILKCD--------------
                                    ---------+---------+---------+---------+---------+---------+---------+---------+
                                            330       340       350       360       370       380       390       400
                                    ---------+---------+---------+---------+---------+---------+---------+---------+
N. risticii Too16                   .............-------------------------------.-----------.....V....--------------    101
N. risticii Ont15                   .............-------------------------------.-----------.....V....--------------     79
N. risticii PA-1                    .............-------------------------------.-----------.....V....--------------    101
N. risticii Cup17                   ----------------------------------------------------------...V....--------------     65
N. risticii Gab17                   ----------------------------------------------------------...V....--------------     65
N. risticii OH07-1                  .............-------------------------------.-----------.....V....--------------    101
N. risticii Reg16                   ---------------------------------------------------------....V....--------------     81
N. risticii Lad17                   .............-------------------------------.-----------..........--------------    101
N. risticii Jan17                   .............-------------------------------.-----------..........--------------     97
N. risticii Luc17                   .............-------------------------------.-----------..........--------------    101
N. risticii May17                   .............-------------------------------.-----------..........--------------    101
MI (US patent)                      E..T.S..KEI.TNEVAKEVLKSDKFKDAITGAGKDALKEILTC.KFKDAVTGNGKDAL....T..--------------    372
N. risticii Illinois                E..TSDKFKK.FE-----------DN--TKAG----YVKEILT-N-----------D......TN.--------------    279
N. sennetsu Miyayama                ------------------------------------------------------------...TS.--------------     82
Neorickettsia sp. from F. hepatica  ....N....DV..-------------------------------.-----------AN.....TSET-------------    107
OR (US patetnt)                     ....S....EV..-------------------------------.-----------AN.....TDQTGKEVLKNSTAKDI    129
```

```
Majority                            ----------------------KFKEAITG-------------------------------------XX--X--AGK---
                                    ---------+---------+---------+---------+---------+---------+---------+---------+
                                            410       420       430       440       450       460       470       480
                                    ---------+---------+---------+---------+---------+---------+---------+---------+
N. risticii Too16                   ----------------------........--------------------------------------------...---   112
N. risticii Ont15                   ----------------------........--------------------------------------------...---    90
N. risticii PA-1                    ----------------------........--------------------------------------------...---   112
N. risticii Cup17                   ----------------------........--------------------------------------------...---    76
N. risticii Gab17                   ----------------------........--------------------------------------------...---    76
N. risticii OH07-1                  ----------------------........--------------------------------------------...---   112
N. risticii Reg16                   ----------------------...D....--------------------------------------------T..---    92
N. risticii Lad17                   ----------------------...D....--------------------------------------------T..---   112
N. risticii Jan17                   ----------------------........D---------------------GKDILKGILTDSTGNFKGAITG...---   131
N. risticii Luc17                   ----------------------........D---------------------GKDILKGILTDSTGNFKGAITG...---   135
N. risticii May17                   ----------------------........D---------------------GKDILKGILTDSTGNFKGAITG...---   135
MI (US patent)                      ----------------------...D.V..NGKDKLKEILTHEKFKALIESEGKDILKGILTDSTGNFKGAITG...---   427
N. risticii Illinois                ----------------------........---------------------DGKDILKEILTDSTGNFKGAITG...---   313
N. sennetsu Miyayama                ------------------------------------------------------------------KFKGLIQSTA.---    93
Neorickettsia sp. from F. hepatica  --------------AKEVLKNS....T...A---------------------GKDVLKEILTDSTGKFKELIES...---   149
OR (US patetnt)                     LKDTNAAEVLKDDTAKEVLKNS........A---------------------GKDILKDILTDSTGKFKELIES...EKV   188

Majority                            --------------------------------------------------------------------------------
                                    ---------+---------+---------+---------+---------+---------+---------+---------+
                                            490       500       510       520       530       540       550       560
                                    ---------+---------+---------+---------+---------+---------+---------+---------+
N. risticii Too16                   --------------------------------------------------------------------------------   112
N. risticii Ont15                   --------------------------------------------------------------------------------    90
N. risticii PA-1                    --------------------------------------------------------------------------------   112
N. risticii Cup17                   --------------------------------------------------------------------------------    76
N. risticii Gab17                   --------------------------------------------------------------------------------    76
N. risticii OH07-1                  --------------------------------------------------------------------------------   112
N. risticii Reg16                   --------------------------------------------------------------------------------    92
N. risticii Lad17                   --------------------------------------------------------------------------------   112
N. risticii Jan17                   --------------------------------------------------------------------------------   131
N. risticii Luc17                   --------------------------------------------------------------------------------   135
N. risticii May17                   --------------------------------------------------------------------------------   135
MI (US patent)                      --------------------------------------------------------------------------------   427
N. risticii Illinois                --------------------------------------------------------------------------------   313
N. sennetsu Miyayama                --------------------------------------------------------------------------------    93
Neorickettsia sp. from F. hepatica  --------------------------------------------------------------------------------   149
OR (US patetnt)                     KALLTDENFKKLFEDDTKANHVKEVLTDINAKEILTDQTAKEVLKDSTAKEVLKHTKFKEAITGAGKDILKDILTDSTGK   268

Majority                            ----------DELKYILTNSEFKSLFDSKDSAEAVKAIFTHSKFKELL
                                    ---------+---------+---------+---------+--------
                                            570       580       590       600
                                    ---------+---------+---------+---------+--------
N. risticii Too16                   ----------...............................N......                                   150
N. risticii Ont15                   ----------...............................N......                                   128
N. risticii PA-1                    ----------...............................N......                                   150
N. risticii Cup17                   ----------.........N............K.......DT...T..                                   114
N. risticii Gab17                   ----------.........N............K.......DT...T..                                   114
N. risticii OH07-1                  ----------.........N......ED.K......E...........                                   150
N. risticii Reg16                   ----------.........N......ED.K..........DT...T..                                   130
N. risticii Lad17                   ----------........SN......ED.K..........DT...T..                                   150
N. risticii Jan17                   ----------..........................E...........                                   169
N. risticii Luc17                   ----------..........................E...........                                   173
N. risticii May17                   ----------..........................E...........                                   173
MI (US patent)                      ----------....D...........E.............NA...G..                                   465
N. risticii Illinois                ----------..........................E...........                                   351
N. sennetsu Miyayama                ----------.K..D....DT..A...EA.T.........DN...G..                                   131
Neorickettsia sp. from F. hepatica  ----------N.F.DL...DS..G...KT...Q.......K.EL.P.V                                   187
OR (US patetnt)                     LKGLIESTGKN.F.DL...DS.......TN..Q.......K.EL.P..                                   316
```
