# Supplementary material for: Isolation and Molecular Analysis of a Novel Neorickettsia Species That Causes Potomac Horse Fever
Source: mBio. 2020 Feb 25;11(1):e03429-19. doi: 10.1128/mBio.03429-19 (PMC7042704; doi:10.1128/mBio.03429-19)
Supplement: TABLE S2 [file mBio.03429-19-st002.pdf]

Table S2. Sequences used in this study

Sample ID	Geographic Location	Fragment size obtained (bp)	Fragment size Compared (bp)	Gene(s) amplified	Accession no. (DNA)
***Neorickettsia* sp. 081**	Ohio, US	761<br>717<br>1498	269, 761<br>513<br>1323	*P51*<br>*Ssa3*<br>*16S rRNA*	AY005443<br>HQ857590.1<br>MK281366
***N. risticii* Ont15**	Ontario, Canada	245<br>387	245<br>384	*P51*<br>*Ssa1*	KX189633<br>KX189634
***Neorickettsia* sp. SF Oregon**	Oregon, US	2417<br>377<br>1332	1392<br>375<br>1323	*P51*<br>*Ssa3*<br>*16S rRNA*	AY376442<br>HQ906699<br>KX462530
***Neorickettsia* sp. SF Hirose**	Japan	2417<br>1435	1392<br>1323	*P51*<br>*16S rRNA*	AY050313<br>U34280.1
***N. risticii* TW2-1**	Pennsylvania, US	259	259	*P51*	AY388494
***N. risticii* MN**	Minnesota, US	676<br>1029	269<br>-	*P51*<br>*Ssa3*	HQ857594<br>HQ857592
***N. risticii* Herodia**	Kentucky, US	673<br>1460	269<br>-	*P51*<br>*Ssa3*	HQ857589<br>HQ857587
***N. risticii* Illinois**	Maryland, US	1538<br>1506<br>1302<br>1498	1410<br>543<br>513<br>1323	*P51*<br>*Ssa1*<br>*Ssa3*<br>*16S rRNA*	WP_015816118.1<br>WP_015816716<br>WP_015816717<br>NR_029162
***N. risticii* Pennsylvania**	Pennsylvania, US	1455	1322	*16S rRNA*	AY005439.1
***N. risticii* Horse 1 (PA-1)**	Pennsylvania, US	1449<br>1448	1407<br>1322	*P51*<br>*16S rRNA*	AF380265<br>AF380257.1
***N. risticii* PA-1**	Pennsylvania, US	765	450	*Ssa1*	HQ857584
***N. risticii* 25-D**		1536	1410	*P51*	U85785
***N. risticii* 90-12**		2573	1407	*P51*	U85784
***N. sennetsu* Miyayama**	Japan	2438<br>999<br>1498	1407<br>-<br>1323	*P51*<br>*Ssa*<br>*16S rRNA*	AY077588.1<br>ABD46359.1<br>NR_074386.1
***N. sennetsu* Kawano**	Japan	1478	1407	*P51*	AY376444
***N. sennetsu* Nakazaki**	Japan	1536	1407	*P51*	AY376443
Neorickettsia* sp. from *F. hepatica	Oregon, US	511<br>344<br>1498	469<br>187<br>1323	*P51*<br>*Ssa1*<br>*16S rRNA*	KYH12366<br>KYH12825<br>LNGI01000001
***Neorickettsia* sp. from Lecithodendridae**	Oregon, US	1332	1323	*16S rRNA*	KF878083
**MI**	Michigan, US	1860	1395	*Ssa1*	US Patent
**OR**	Oregon, US	1422	948	*Ssa1*	US Patent
