# Supplementary material for: Isolation and Molecular Analysis of a Novel Neorickettsia Species That Causes Potomac Horse Fever
Source: mBio. 2020 Feb 25;11(1):e03429-19. doi: 10.1128/mBio.03429-19 (PMC7042704; doi:10.1128/mBio.03429-19)
Supplement: TABLE S3 [file mBio.03429-19-st003.docx]

# Table S3. Putative outer membrane proteins of *Neorickettsia* spp. ^[[1]](#footnote-1)^

| Fin17 GenBank Accession No. ^[[2]](#footnote-2)^ | Gene Symbol | Protein Name | MW (kDa) [aa number] | | |
| --- | --- | --- | --- | --- | --- |
|  |  |  | **Fin17** | **NRI** | **NSE** |
| P51 Protein: | | | | | |
| WP_015816118 (-) | *p51* | P51 gram-negative porin family protein | 54.3 [505] | 54.8 [512] | 54.5 [511] |
| NSP Proteins: | | | | | |
| WP_015816683 (+) | *nsp1* | *Neorickettsia* surface protein 1 | 27.6 [255] | 28.0 [260] | 27.9 [261] |
| WP_015816684 (+) | *nsp2* | *Neorickettsia* surface protein 2 | 33.5 [308] | 33.5 [308] | 33.2 [304] |
| WP_015816686 (+) | *nsp3* | *Neorickettsia* surface protein 3 | 27.1 [260] | 25.5 [242] | 26.2 [246] |
| SSA Proteins: | | | | | |
| WP_160095937 (+) | *ssa1* | Strain-specific surface antigen 1 | 51.5 [473] | 54.5 [501] | 29.4 [265] |
| WP_160095941 (+) | *ssa2* | Strain-specific surface antigen 2 | 54.3 [487] | 56.8 [512] | 7.4 [66] |
| WP_015816717 (-) | *ssa3* | Strain-specific surface antigen 3 | 47.6 [434] | 47.4 [433] | 36.4 [332] |

1. Putative OMPs (P51, NSP1/2/3, and SSA1/2/3) are determined by homology searches to *N. risticii* and *N. sennetsu* protein database using BlastP. Fin17, *Neorickettsia* sp. Fin17 (data obtained from in this study); NRI, *N. risticii* Illinois; NSE, *N. sennetsu* Miyayama. Ssa2 protein of NSE is degenerate. [↑](#footnote-ref-1)
2. Coding strands for each OMPs were indicated in parenthesis: +, forward strand; -, reverse complementary strand. [↑](#footnote-ref-2)
